# Monoclonal antibodies: From magic bullet to precision weapon

**DOI:** 10.1186/s43556-024-00210-1

**Published:** 2024-10-11

**Authors:** Hassan Aboul-Ella, Asmaa Gohar, Aya Ahmed Ali, Lina M. Ismail, Adham Ezz El-Regal Mahmoud, Walid F. Elkhatib, Heba Aboul-Ella

**Affiliations:** 1 Department of Microbiology, Faculty of Veterinary Medicine, Cairo University, Giza, Egypt; 2 Department of Microbiology and Immunology, Faculty of Pharmacy, Galala University, Suez, Egypt; 3 Department of Microbiology and Immunology, Faculty of Pharmacy, Sinai University, Sinai, Egypt; 4 Department of Biotechnology and Molecular Chemistry, Faculty of Science, Cairo University, Giza, Egypt; 5 Department of Biotechnology, Faculty of Agriculture, Ain-Shams University, Cairo, Egypt; 6 Department of Pharmacognosy, Faculty of Pharmacy and Drug Technology, Egyptian Chinese University (ECU), Cairo, Egypt; 7 Department of Microbiology and Immunology, Faculty of Pharmacy, Ain Shams University, Cairo, Egypt; 8 Scientific Research Group in Egypt (SRGE), Cairo, Egypt; 9 Department of Microbiology and Immunology, Faculty of Pharmacy, Ahram Canadian University (ACU), Giza, Egypt; 10 Egyptian Drug Authority (EDA), Giza, Egypt; 11 Creative Egyptian Biotechnologists (CEB), Giza, Egypt

**Keywords:** Magic bullet, Monoclonal antibodies (mAbs), Immunotherapeutic, Next-generation mAbs, In vitro display, AI-assisted mAbs development

## Abstract

Monoclonal antibodies (mAbs) are used to prevent, detect, and treat a broad spectrum of non-communicable and communicable diseases. Over the past few years, the market for mAbs has grown exponentially with an expected compound annual growth rate (CAGR) of 11.07% from 2024 (237.64 billion USD estimated at the end of 2023) to 2033 (679.03 billion USD expected by the end of 2033). Ever since the advent of hybridoma technology introduced in 1975, antibody-based therapeutics were realized using murine antibodies which further progressed into humanized and fully human antibodies, reducing the risk of immunogenicity. Some benefits of using mAbs over conventional drugs include a drastic reduction in the chances of adverse reactions, interactions between drugs, and targeting specific proteins. While antibodies are very efficient, their higher production costs impede the process of commercialization. However, their cost factor has been improved by developing biosimilar antibodies as affordable versions of therapeutic antibodies. Along with the recent advancements and innovations in antibody engineering have helped and will furtherly help to design bio-better antibodies with improved efficacy than the conventional ones. These novel mAb-based therapeutics are set to revolutionize existing drug therapies targeting a wide spectrum of diseases, thereby meeting several unmet medical needs. This review provides comprehensive insights into the current fundamental landscape of mAbs development and applications and the key factors influencing the future projections, advancement, and incorporation of such promising immunotherapeutic candidates as a confrontation approach against a wide list of diseases, with a rationalistic mentioning of any limitations facing this field.

**Supplementary Information:**

The online version contains supplementary material available at 10.1186/s43556-024-00210-1.

## Introduction

Antibodies are one of the naturally existing and primary pathways through which the body defends itself against antigens, which may be derived from bacteria, viruses, fungi, parasites, bacterial/virus-infected cells, pollen, or nonliving substances, such as toxins, chemicals, drugs, or foreign particles considered alien to the body as epitopes expressed on cancer cells, etc. Specific binding to their targets is consequenced by either neutralizing and interfering with their pathogenic effect or flagging them for clearance or destruction as one of the Ag-Ab immune-complex fates. Antibodies are considered one of the most prominent and promising remarks in the medicinal, pharmaceutical, and even veterinary fields, with a wide range of significant diseases’ prophylactic, therapeutic, and diagnostic approaches [1–9], on the cusp of the modern medicine era. The natural development of antibodies within a living creature’s body due to active infection are polyclonal antibodies (pAbs), derived from several B cell clones’ development due to the nature of our extremely diverse B cell repertoires [10, 11].

On the other hand, the mAbs can bind specifically to more concise and specific certain antigen-functionally essential epitopes to which they were primarily developed. They can also be engineered to optimize their binding, functional activity, or half-life period to be more attributable to the global therapeutics markets. Extreme progress has evolved through the introduction of mAbs to non-communicable disease therapy [12], such as cardiovascular disease (CVD) [13, 14], chronic respiratory syndromes (CRS) [15], autoimmune diseases [16, 17], diabetes [18, 19], which are the most incriminated causes of global mortality, elevated lower density lipoprotein (LDL) level [20], and cancers [21–34]. The introduction of mAbs in the cancer treatment protocols, more than 100 years ago, greatly transferred some of the difficult-to-be-cured cancers to the treatable list and millions of people around the world now live better or even have their lives spared owing to cancer immunotherapy [19, 21, 23, 35].

The development of therapeutic antibody agents to treat infectious diseases lags far behind that of those to treat non-infectious diseases, although it is well-known that antibodies can control infectious diseases [36–39]. In comparison to the reagents used in immunotherapies, antibiotics are less costly and simpler to make. Therefore, Antibiotics have been given priority in the treatment of bacterial infectious disorders [40, 41]. Furthermore, until the process of infection between a virus and its host cell is understood, it is frequently impossible to design a strategy for the development of antibody medicines for viral infectious disorders [38]. To parasitize and multiply, viruses infiltrate host cells, making it challenging for the host immune system to identify them as foreign enemies. Furthermore, in certain viral infections, vaccination can sometimes make symptoms worse because of a process called antibody-dependent enhancement (ADE) [42]. In this scenario, the virus binds to suboptimal antibodies, which increases the virus's ability to enter host cells [43].

**Fig. 1:**
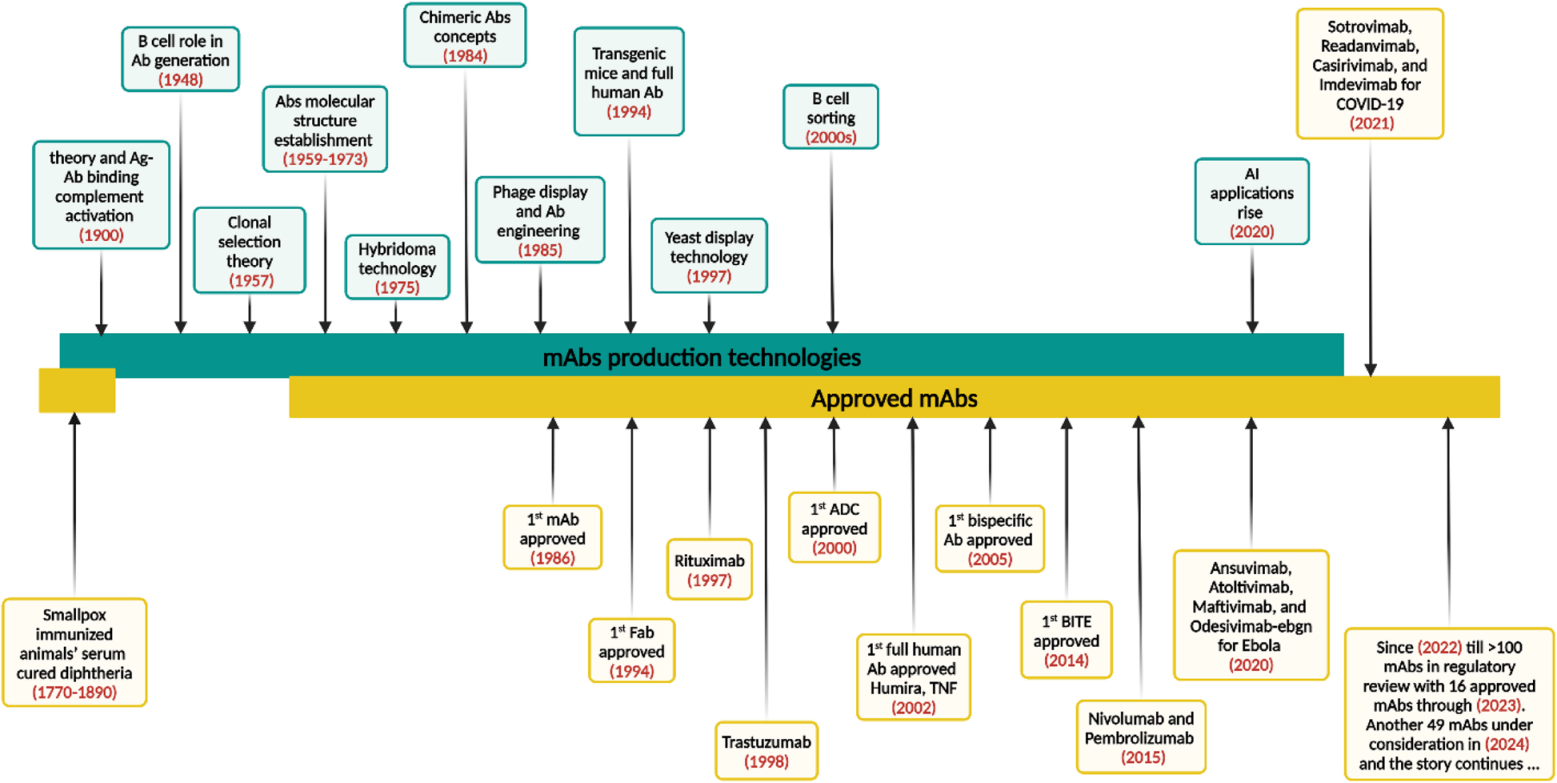
An illustrative diagrammatic presentation of the overall journey and the shiny remarks of the mAbs development technologies, advancement, and the most prominent approved mAbs

The COVID-19 pandemic and the emergence of antimicrobial-resistant micro-organisms have drawn renewed interest in human mAb therapies in recent years [44, 45]. Furthermore, compared to attenuated or inactivated vaccines or immunoglobulin preparations, the administration of human mAb as therapy against infectious diseases is far safer [46]. Moreover, the development of human-neutralizing mAbs against infectious diseases has improved with technological advances in human immunoglobulin transgenic mice, single cell B cell receptor (BCR) DNA sequencing, and phage display [38]. And many successful cases have been reported in the past decade, Jimmy Carter, a former United States (US) president, is only one well-known success tale. He was diagnosed as being disease-free after receiving pembrolizumab for metastatic melanoma [47]. As well as former US President Donald Trump received REGN-COV2, Regeneron Pharmaceuticals’ experimental mAb cocktail, as part of his treatment protocol for COVID-19 [48]. All are shiny remarks in the history of using mAbs in the field of therapeutics and pharmaceutics-safe alternatives Fig. 1.

In this context, this review aims to comprehensively: (1) highlight the fundamentals of mAbs-development and optimization technologies, (2) focus on the wide range of current therapeutic applications of mAbs in non-communicable diseases, (3) shed light on the ongoing development of mAbs-dependent antimicrobials for communicable diseases, (4) clearly describe the limitations and possible adverse reactions associated with the use of mAbs, and (5) in a prospective manner mentioning prospective insights on incorporating artificial intelligence (AI) to accelerate and redirect mAbs development and applications. Also, the hierarchal structure of the current work is provided in a diagrammatic illustration in Supplementary Fig. 1.

## Fundamentals of mAbs’ development and optimization technologies

**Fig. 2:**
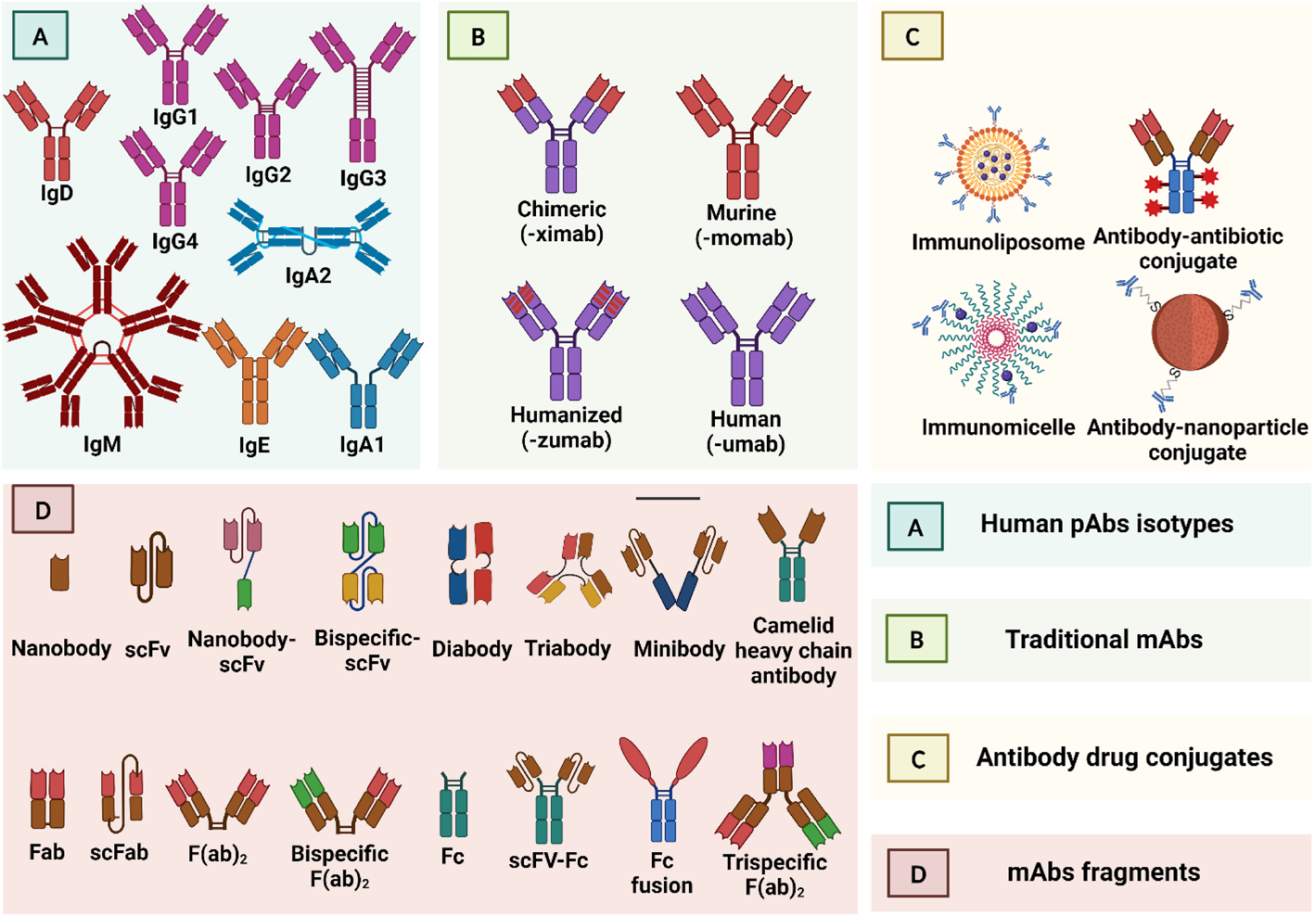
A detailed diagrammatic illustration of the distinctive structural similarities and differences between all known categories and types of antibodies

The most common therapeutic antibody modalities are human and humanized mAbs; 51, 34.7, 12.5, and 2.8% of all mAbs in clinical use are for human, humanized, chimeric, and murine antibodies, respectively [49]. The next part covers the standard mAbs’ production platforms with a detailed description of their basic and supplementary techniques. They are followed by an illustrative mention of the basis of the optimization and maturation of mAbs binding affinity. Lastly, the wide range of next-generation mAbs and the basics of their developing techniques will be discussed while focusing on the structural differences and similarities between each known mAb Fig. 2.

### Standard mAbs’ production platforms

#### Mouse hybridoma

**Fig. 3:**
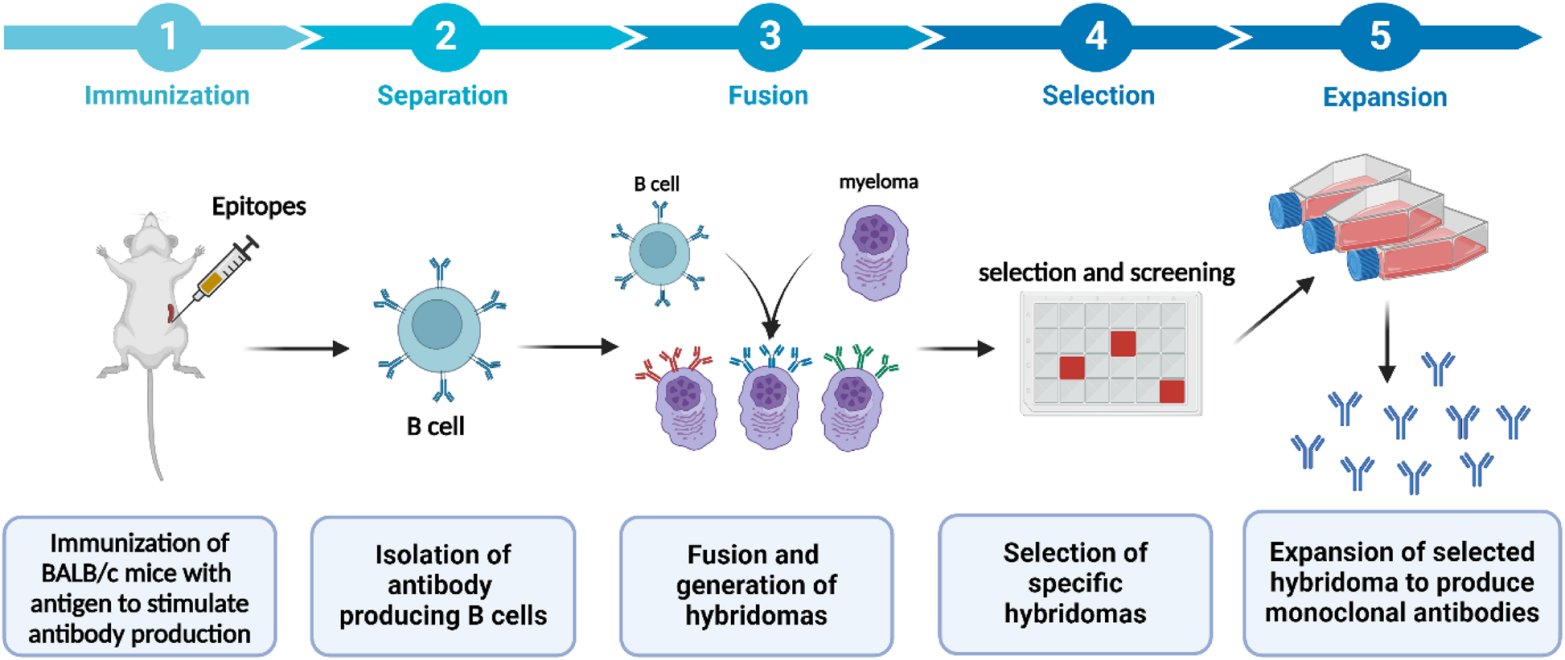
Illustrative diagram for the general flow and major steps of the hybridoma technology

Murine mAbs have several advantages, such as their wide availability, low cost, and quick production time Fig. 3, making them highly competitive for large-scale adoption. However, the use of non-humanized murine mAbs in therapeutic settings has been associated with various documented drawbacks. When patients receive mouse mAbs, they quickly develop human anti-mouse antibodies (HAMA). These HAMAs can also lead to unwanted hypersensitivity reactions. Additionally, patients' response to murine fragment crystallizable region (Fc) by antibody-dependent cellular cytotoxicity (ADCC) initiation is limited. In contrast, humanized mAbs can resolve the immunogenicity problems of murine antibodies and are more efficient in carrying out effector functions [50].

In humanized mAbs, only the heavy and light chains' complementary-determining regions (CDRs) are of murine origin. The development of humanized mAbs began in 1988 [51–53], and one of the most well-known techniques for producing them is CDR grafting, pioneered by Gregory P. Winter in 1986 [54]. This technique guarantees the antibody's ability to bind to the target antigen by inserting CDR sequences of non-human origin into human framework sequences. Daclizumab was the first Food and Drug Administration (FDA)-approved humanized monoclonal antibody in 1997, which was grafted with CDRs and used to prevent transplant rejection by binding to IL-2 receptors [55]. Furthermore, by "human back to mouse" mutations in CDR-grafted humanized antibodies, the locations of amino acid residues in the framework may subsequently be taken into consideration for restoration, enhancing the stability and affinity of the finished product.

To support humanization research, integrated bioinformatics, and antibody structure databases are presently being constructed on web servers. [56–59]. They provide materials for modeling antibodies, grafting, evaluating back mutations, and selecting human templates. However, if the antibodies' ability to attach is still compromised, more affinity maturation needs to be done. To assess how human the variable area of mAbs is, numerous methods have been developed. The "H-score" technique was developed by Abhinandan and Martin to evaluate antibody sequences' "degree of humanness". The mean sequence identity is computed by comparing it to a subset of the database of human variable region sequences. [60]. A germinality indicator was developed to facilitate the germline humanization of a macaque antibody [61]. The G-score was created from the H-score to better classify the germline framework sequence [62].

A large library of about 38,700 human antibody variable region sequences served as the foundation for the T20 score analyzer, which made it simple to differentiate human sequences from mouse sequences and those of many other species. It drew attention to the parallels between fully human and humanized antibodies [63]. These web-based humanness calculators make it possible to support the production of humanized antibodies. The development of humanized antibodies has significantly improved the clinical tolerability of mAb treatments. Making mAbs for a range of conceivable medicinal applications has been made feasible by the ability to precisely alter antibody sequences. Presently, half of all mAbs used to treat people are chimeric or humanized mAbs. One of the most well-known humanized antibodies, trastuzumab, was approved in 1998 and sold more than 7 billion dollars annually in 2018. Trastuzumab is used to treat patients with gastroesophageal junction adenocarcinoma and metastatic breast cancer that has progressed to other regions of the body [64, 65].

#### In vitro display of mAbs

The cell and cell-free display technologies presented below have enabled the rapid discovery of novel antibody fragments entirely in vitro. Phage display remains the most widely used in vitro display technology due to its ease of use and versatility. The mammalian display offers several advantages, such as post-translational modifications, that are lacking in other display platforms. Ribosome display allows for cell-free synthesis of antibodies, while yeast display allows for native expression of antibodies. Bacterial display, although less commonly used, offers advantages such as low cost and ease of expression. Also, plant display is another nonmammalian display providing an easy to be expanded and scaled up options compared to other well-established ones.

##### Phage display

The idea of putting external proteins on the surface of phages was initially presented by [66] in 1985, demonstrating the possibility of creating phage libraries with a wide variety of protein repertoires. This approach has been most successfully applied in antibody display libraries. The underlying principle of display technology is that desirable qualities can be selected from a big library of antibodies after they are generated [67]. An array of billions of phages with various antibodies on their surface can be used to isolate a phage with a particular antibody on its surface and test its ability to bind to a target ligand [68–70]. The choice of a virus permits the simultaneous recovery of the matching antibody gene since the phage genome contains the phage-displayed protein gene [71–80]. DNA sequencing makes it simple to identify genetic features once they are extracted, and the sequence may be employed in other contexts. To carry out this procedure a few essential steps are required.

Initially, the antibody DNA sequences are assembled into a library. The variable regions (VH and VL) are the only areas where antibody variability is present. These gene segments are put into a particular vector such that the sequence encoding the phage protein pIII is in a frame. Eventually, put together, the phage particle will reveal the functional antibody fragment that has been fused to the minor coat protein III's amino terminus. When building an antibody library, there are various options to consider: (1) What kind of antibody fragment should be used? (2) Where can I find the repertoire of V regions? The single-chain fragment variables (scFvs) format, which consists of VL and VH segments connected by a flexible linker, or the fragment antigen binding (Fab) format, in which VH-CH1 and VL-CL associate non-covalently has generally been used in effective efforts [69]. Before selecting, it is necessary to evaluate the library's clonal diversity, whether it is synthetic or naïve.

Nowadays, it is common practice to quantify diversity and verify the stated library design using next-generation sequencing. After a library has been established, immobilized, or tagged antigens are used in "phage panning", a process that enriches phage antibodies specific to a particular antigen [81–83]. In the final phase, an enzyme-linked immunosorbent assay (ELISA) evaluation is used to identify specific antibodies for a given antigen within several randomly selected clones. Since antibody genes can now be instantly recognized via gene sequencing, they can undergo further genetic manipulation to achieve specific goals, such as constructing whole immunoglobulins alongside the intended effector properties or increasing affinity by creating transformed antibody secondary libraries [84].

##### Yeast display

The yeast surface display method was created by Boder and Wittrup in 1997 [85]. Antibodies used in yeast display are usually expressed as scFvs, Fabs, and even full-length Igs (IgG1 [86] and IgGs [87]) in yeast cells, most frequently *S. cerevisiae*. Usually, the two proteins, Aga1 and Aga2, are used as anchors between the yeast cell and the antibody fragment [88]. Different methods can be used to select antibody fragments; commonly, fluorescence-activated cell sorting (FACS), magnetic bead-based selection, and NGS-based methods are used. One method of achieving affinity maturation is bio-panning. Yeast display has certain benefits over phage display, such as being suitable for in vivo applications and expressing antibodies in their original form. On the other hand, yeast display libraries are generally smaller.

##### Mammalian display

Mammalian cell display is a more recent technique for creating antibody libraries and choosing particular antibodies. This approach uses mammalian cells to generate antibodies as fusion proteins, which may then be chosen using a variety of methods such as FACS, magnetic bead-based selection, and bio-panning. Native form expression of antibodies is one of the primary benefits of mammalian display. Since the method can display full-length Ig molecules on the cell surface, it can also be used in production. One of the biggest limitations of the approach is the small size of mammalian display libraries; nonetheless, mammalian cells have been shown to display quite large antibody libraries. For example, [89] designed an IgG library of 10^7^ CDR3 mutated clones for affinity maturation to identify antibody clones with better PD-1-blocking properties.

##### Bacterial display

In bacterial display, Antibody fragments can be shown on the surface of bacterial cells, lipoproteins like Lpp-OmpA are frequently used in this process. *E. Coli* is one of the frequently utilized bacteria in peptide display [90]and epitope mapping [91], where it is used to display antigens. Furthermore, *E. Coli* [92, 93] and Gram-positive Staphylococci; *S. xylosus* [94], *S. aureus* [95], and *S. carnosus* [96] have been employed for antibody display. Fragments, VHH, and full-length IgGs are among the formats shown. The disadvantages of the display approach include reduced transformation efficiencies (for Gram-positive bacteria) and post-translational changes that differ from those in human cells. However, bacterial cells are a viable option for in vitro display due to their vast library sizes and rapid growth rates.

##### Ribosome display

Ribosome display is a cell-free display technology that eliminates the need for living cells in the development and selection of antibodies. Rather, it uses processes for cell-free protein synthesis to make and present antibody fragments. For ribosome display, display techniques for big, varied libraries of multi-chain fragments for selection and display have been created. Fab fragments could even be produced as full-length IgGs according to a technique described by [97]. Large libraries of up to 10^12^ and 10^15^ antibody variants are possible because of ribosome display, which is not constrained by the efficiency of host cell transformation. Because no cell culture is needed, it is also quick. One technological drawback is that fewer functional ribosomes are accessible [98].

##### Plant display

The expression of at least two polypeptide types, their appropriate assembly into a multimeric structure, and complex-type glycan modifications are necessary for the creation of antibodies. Since the first mAb was produced in tobacco in 1989, plants have been demonstrated to be able to manufacture mAbs despite this complexity [99]. Since then, a wide range of mAbs and their structural variants have been produced, some of which have entered human clinical trials [100–103]. These variants include IgGs, secretory IgAs, pentameric IgMs, camelid nanobodies, tetravalent mAbs, bifunctional mAbs, recombinant immune-complex (RIC), single-domain fragments, scFvs, and diabodies. Plant-based systems to produce monoclonal antibodies are characterized by their low cost, excellent scalability, and little danger of human pathogen contamination [104–106].

In contrast to mammalian cell culture techniques, simple mineral solutions can be used in greenhouses to grow plant biomass. This results in significant cost reductions related to upstream processing of mAb synthesis, since it does away with the requirement for capital-intensive bioreactors and costly growth media [100, 107]. Drugs may become contaminated with animal diseases during the production of mAbs in mammalian cells, particularly if the pathogens are unidentified or poorly understood. Plant-produced mAbs significantly lower the risk of infection since human-infecting pathogens are rarely found in plants. Plant cells have an additional benefit for creating mAbs and may provide a novel avenue for drug delivery due to the ease with which many hetero-subunit proteins may be produced and the distinct properties of the cell wall.

Plants have been successfully used to manufacture pentameric IgMs that require up to four hetero-subunits and functional, protease-resistant secretory IgAs [101, 108]. These mAbs can enter the gut lumen and be enzymatically released by commensal bacteria there because they are encapsulated inside the plant cell wall, which shields them from the stomach's acids and enzymes [109]. This provides the opportunity to administer edible plant materials encapsulating mAbs against gastrointestinal (GI) viruses orally to treat GI viral disorders. Plant-cell-supplied mAbs can be utilized as prophylactics to limit viral entrance and as treatments to treat viral infection because many viruses employ mucosal surfaces as sites of entry. Large volumes of mAbs would be required if prophylactic mAb applications were used to prevent viral infections since they would enroll bigger groups with recurrent doses. Because plant-based production platforms can easily scale up production without requiring prohibitive capital expenditure or the time-consuming development of new production processes, they become more desirable in this situation [100].

Simply, the overall difference points between the different display technologies can be briefly described and summarized in Table 1.

**Table 1:** A brief tabulation of the main difference points between the different in vitro display techniques of mAbs

In vitro display technology	Brief description	Reference
Phage display	Antibody fragments that were displayed on the surface of bacteriophages made it possible to quickly and efficiently select and optimize high-affinity antibodies	[66–84]
Yeast display	Yeast display provides a high-throughput platform for antibody discovery and optimization to express and display antibodies. It is possible to show fragments or whole-length antibodies	[85–88]
Mammalian display	Mammalian cells are utilized in antibody display and expression. enables the proper folding and presentation of full-length antibodies or antibody fragments with post-translational modifications	[89]
Bacterial display	Appearance of antibody fragments or even complete IgGs on the bacterial outer or inner membrane. Numerous approaches, and quick development rates. Gram-positive bacteria can produce post-translational modifications (PTMs) that are not human	[90–96]
Ribosome display	A cell-free display device that generates and shows antibody fragments using cell-free protein synthesis processes, not constrained by the efficacy of host cell transformation. Full-length IgG may be displayed	[97, 98]
Plant display	Beyond the well-known advantages of cheap cost and high scalability, current plant-based technologies offer several advantages for the research and manufacture of mAb. To fight present and upcoming pandemics, novel expression vectors have made it possible to produce mAbs at high numbers at a speed never before possible. Plants are now able to generate mAbs with distinct mammalian glycoforms and a high level of homogeneity thanks to host glycoengineering	[99–109]

#### Transgenic mouse

Transgenic mice offer a reliable platform for the development of antibody-based medications. Compared to traditional approaches, transgenic animals offer numerous advantages in the production of human antibodies, such as the removal of the need for humanization, increased diversity, in vivo affinity maturation, and clonal selection for antibody optimization. However, the development of transgenic mouse antibody technology was complicated by the size of human Ig loci. Furthermore, a variety of rearrangements as well as robust expression of the human V, D, and J segments are needed to build repertoires in transgenic mice that are close to or comparable to those in humans [110].

Several methods have been employed to produce animals with human antibody repertoires to address these significant obstacles [111–113]. The first suggestion for the feasibility of producing human antibodies in transgenic mice was made in 1985 by [114], who suggested introducing human antibody genes into the mouse germline. This creative innovation gave the production of human antibodies a new direction. In 1989, cloning the first human heavy chain was successfully constructed [115]. In 1992, [116] successfully cloned the human kappa light chain construct, which consists of one human kappa light chain variable (V) gene, a human kappa light chain joining cluster (J), and a human kappa constant region (C). The expression of mouse endogenous Ig was incompatible with the expression of human antibodies, despite the mice expressing human kappa light chain and human heavy chain (VH-D-JH-C-C1).

Less than 10% of all antibodies are human antibodies. Concurrently, many murine Ig knockout mouse strains were produced. In 1993, Chen and colleagues knocked off the murine JH and J genes via gene-targeted deletion [117–124]. Despite the creation of this line leading to the growth of human Ig genes and the eradication of mouse endogenous Ig interference, human antibody production, Ig class switching, and somatic hyper-mutation are still less effective than mouse constant region gene expression [125].

#### Single B cell mAbs

Regarding therapeutic applications, human B cell-derived mAbs are superior to alternative technologies like phage display in several ways. They exhibit a relatively high selectivity towards their targets, often proteins, because of their in vivo growth and affinity maturation. This allows for a customized response with minimal off-target binding to other host proteins. In keeping with the first trait, they often exhibit modest immunogenicity when administered in vivo. Therefore, when compared to mAbs obtained from, say, phage display libraries, hybridoma-derived mAbs have, on average, had a better general developability profile. This critical factor is thought to constitute the "Achilles heel" of most biologics. Their native post-translational modification profile also contributes to this, as it reduces the likelihood of aggregation both in vitro and, crucially, in vivo [126].

Reliable PCR-based amplification of VH and VL genes, along with their pairing and sequencing, is a crucial step in recovering antibody-coding sequences from single B cells. There are numerous degenerate primers and primer sets (often for nested PCR) that are available for the most frequently studied species, particularly mice and humans. Based on the up-to-date knowledge, the primer sets released have shown promising results that enable reliable amplification of murine and human Ig genes [127–129].

##### Identification and isolation of single B-cell

Single B cell isolation from PBMCs or lymphoid tissues can be achieved by FACS [130–134], micromanipulation [130, 131], or laser capture microdissection [132]. Usually, bone marrow or PBMCs are utilized to separate mononuclear cells using Ficoll-Paque density gradient centrifugation. When identifying rare and unique B cell subpopulations, FACS is often employed to separate individual B cells based on the expression of cell surface markers by B cells at different phases. Antigen-coated magnetic beads [135] and fluorescence-conjugated antigens [136–139] are also used to select antigen-specific B cells in a process called "antigen baiting". Neutralizing human mAbs and antigen-coated magnetic beads were used to harvest B cells. Commonly, antigen-specific B cells have been identified using antigen-conjugated fluorescent beads [140]. Fluorescent rotavirus-like particles were used as antigen bait to excite single RV-specific B lymphocytes that were isolated from healthy rotavirus-infected infants or adult donors [141]. HIV envelop protein antigens have also isolated antibodies that broadly neutralize HIV-1 [142, 143]. Furthermore, it was reported that dengue virus-specific memory B cells have been identified [144, 145]. For a polyclonal mixture, antigen baiting can therefore be used as an initial selection approach.

##### Cloning of single B-cell and screening of mAbs

After sorting a single B cell, each Ig heavy chain and matching light chain should be directly cloned [133, 134]. This stage involves amplifying each identified B cell's distinct heavy and light chains via nested or semi-nested reverse transcription-polymerase chain reaction (RT-PCR) methods. Reverse primers target the constant fragments, and forward primers target the variable fragments [133, 146]. Various primer-set combinations can be optimized to improve the recoveries of the VH and VL [133, 147]. After that, the genes are cloned and expressed in mammalian cell lines to produce recombinant mAbs. Techniques for a cell-based microarray chip method [148] and a micro-engraving method [149–152] have also been reported for high-throughput screening and assessment of secreted mAbs with ideal reactivity.

Certain antibody-secreting cells can be identified and recovered using the immunospot array assay on a chip, a cell-based microarray chip technique that enables the trapping of secreted antibodies by a chip coated in antibodies against Ig [148]. The procedure of micro-engraving, which produces microarrays containing the secreted antibodies of individual cells, uses a soft lithographic technique [149]. The early and rapid identification of high-affinity and specificity clones of the target antigen is an advantage of these two techniques.

#### B cell immortalization

Human B cell clones that have been immortalized are thought to be the best source of mAbs [153]. Epstein-Barr Virus (EBV) transformation is often used to induce human B cell immortalization. Normal human B cells are transformed into established lines by the lymphotropic herpes, EBV. The properties of the original B cell, including surface and secretory Ig and EBV and complement receptors, are retained through this process of "immortalization". Normal lymphocytes are activated by EBV transformation, which also causes secretory Ig to be released. It has recently been demonstrated that the addition of a Toll-like receptors (TLR) agonist can boost the modest efficiency of B cell immortalization (0.1%) [154]. This approach helps find uncommon cells that produce different Abs due to its high-throughput capacity and ability to screen functional Abs directly [155]. 

This strategy's brief window of opportunity to find the required Ag-specific clone and B cells' transient growth are its drawbacks. Retrovirus-mediated gene transfer has also been utilized to immortalize B cells in addition to EBV. B cells are transduced using retroviruses containing BCL-6 and BCL-XL after they have been identified and activated by specific individuals. In the presence of IL-21, transduced B cells proliferate on fibroblasts that express CD40L. The selection of B cell clones is based on secreted Abs and the ability of the clones to produce the required Abs is tested [156]. A practical and effective technique for creating a genuine human Ab library, a crucial resource for creating human mAbs. More research is needed to enhance this technology because the production of mAbs based on human B cell immortalization is still limited to preliminary stages due to several challenges associated with its methodology, which need to be overcome by further research [157].

#### Nucleic acid-encoded mAbs

##### DNA-encoded

The DNA-encoded mAb approach delivers genetic constructs expressing the desired mAbs within the host cells, eliminating the need for customary manufacturing and purification processes. For the effective delivery of mAb, viral vectors, particularly adeno-associated virus (AAV), have been employed [158]. AAV does not integrate into the DNA of the host cell; it can infect both dormant and actively growing cells. Its advantages include comparatively lower immunogenicity and long-lasting genetic cassette persistence following a single injection. However, AAV can trigger immune reactions, which introduces an extra layer of complication in the form of antivector immunity. AAV capsid-neutralizing Abs have the potential to block transduction and the subsequent administration of the same gene therapy vector [159]. Alternatively, DNA plasmids can be used to transfer required genes, enabling in vivo production of mAbs.

Through the process of adaptive electroporation, cells are subjected to a short electrical field pulse that punctures the cell membrane, allowing for the synthesis of mAb after DNA electrophoresis and absorption [160]. Better gene insertion ability, immunity to the vector being avoided, and repeated delivery are among the benefits. Additionally, DNA is more stable at room temperature and has less immunogenicity [161]. Lastly, the ease with which DNA may be produced in huge amounts and the removal of the necessity for logistics of transit and temperature-controlled storage make this approach economical. One of the disadvantages is that it often takes 7 to 14 days to attain maximal expression, which can be too lengthy for therapies to start working right away. The expression is then visible for two to three months. Even if they have not yet been observed in clinical settings, the theoretical dangers of DNA-based therapies, such as insertional mutagenesis and the beginning of immunological reactions against DNA after repeated administration, need to be investigated [162].

##### RNA-encoded

In vitro*,* transcription of Ab-encoding mRNA from a DNA template is required to generate mRNA-encoded mAbs. RNA polymerase-mediated transcription of a linear DNA template having a promoter, 5′ and 3′ untranslated regions, and an open reading frame is the first step in the production of mRNA. The mRNA needs a polyadenylation tail and a triphosphate cap at the end to be biologically active. The delivery of mRNA to the cytosol triggers the synthesis of the encoded mAbs, which then undergo post-translational modifications. This process is completed in a matter of hours, and mRNA-encoded mAbs reach their peak expression within a few days. The genetic information transmitted by mRNA is transient, lasting only until the mRNA is degraded. Depending on the mRNA dose, the properties of the mRNA molecule, and the mechanism used for mRNA administration, protein expression can last from hours to days [163].

The benefits include fast protein expression in just a few hours, enabling repeated dosage schedules. The process supports scalable production of clinical grade mAb batches. This technology exhibits a high clinical safety profile, and its cell-free nature streamlines manufacturing procedures and lowers costs [164]. Because mRNA-encoded mAbs are unstable within cells, carrier platforms must be used to enable effective delivery and expression. Lipid nanoparticles are currently the most adaptable and effective method for delivering mRNA-encoded mAbs [165]. The fact that mRNA-encoded mAbs can be administered only i.v. and that the liver is their primary target organ represents a significant disadvantage [166]. Unaltered mRNAs can generate a cytokine storm inside the body, and mRNA or by-product mRNA transcription can activate the innate immune system through pathogen-associated molecular patterns. Research is needed for efficient purification of mRNAs on a large scale to overcome difficulties associated with transport and immunogenicity [167].

### Optimization and maturation of mAbs binding affinity

Antibodies produced by phage, transgenic, or humanized techniques are frequently modified further, such as by substituting binding liabilities for residues in the binding area. Furthermore, certain point mutations in the structure of the antibody can produce products with higher affinity for the antigen than the original antibody, whereas other mutations will produce lower affinity. Affinity maturation is the process of increasing affinity for antigens. Helper T cells aid in the affinity maturation of mature B cells following V(D) J recombination. The humoral immune response's affinity maturation, which can produce antibodies with low picomolar affinity, is a crucial feature [168, 169]. Antibodies with high affinity are essential for neutralizing cytokines or growth factor-induced signaling. A mAb typically needs to have an affinity of 1 nm or less for the target antigen to be taken into consideration for therapeutic drug development [170].

Furthermore, affinity is typically decreased by humanizing mice mAbs [171]. As a result, affinity maturation is frequently required in the creation of antibody-based drugs [172]. Due to their ease of screening for high-affinity variants and high throughput applications, phage display, and yeast display have been frequently employed for affinity maturation of antibodies [173]. The strategies for boosting antibody affinity fall into two main types. The initial step is to create a sizable library of randomly modified CDR or whole variable domain sequences. From this massive mutant pool, the stronger affinity variants are then chosen. A different strategy is to create tiny libraries via hotspot or targeted mutagenesis, which imitates in vivo affinity maturation. Using a focused approach, a high-affinity variation is chosen and randomized at discrete points in the variable domain known as the hotspot or at sites in each of the six CDRs. Typically, various mutations are combined to produce marginally higher affinity.

The antibody's affinity for the antigen may rise significantly because of the additive or synergistic effects of these several alterations combined [174]. The phage display method can be used in strict bio-panning settings (lower antigen quantity, longer incubation, rigorous washing steps, or competition with soluble antigens) to find high-affinity antibodies in an antibody gene mutation library [149, 175]. The first stage of affinity maturation in vitro is the diversification of antibody genes, which can be accomplished by a variety of techniques including chain shuffling, targeted mutations, and random mutations [175–179]. Since mutations accumulate more quickly in the CDR than in framework residues, this approach is more applicable to somatic mutations that occur in vivo during the evolution of B cells.

### Next generation mAbs

The latest and most recently produced antibody category with fragmented structure and extra unique features.

#### Polyspecific (multispecific) antibodies

##### Bispecific antibodies (bsAbs)

The strong affinity and strict specificity for target antigen antibodies containing two binding sites targeted at two distinct antigens or epitopes on a single antigen is known as bispecific antibodies (bsAbs). Compared to mAbs, bsAbs have better clinical therapeutic outcomes and are widely used in tumor immunotherapy and other medical conditions. Recent advancements in recombinant DNA technology and antibody or protein engineering have led to establishing a few platforms to produce diverse bsAb types based on cutting-edge techniques and intended applications. BsAbs based on the heterologous recombination of heavy chains and matching of light chains have been developed and produced using more than thirty established commercial technological platforms.

With these many kinds of bsAbs, the precise mechanisms of clinical/therapeutic activity have been shown. More than 110 different varieties of bsAbs are undergoing various phases of clinical studies, with three of them having been approved for use in the market. We expand on the traditional platforms, workings, and uses of bsAbs in this paper. Our goal is for this evaluation to improve existing clinical approaches and spark fresh ideas for the development of bsAbs [180].

##### Tri-specific antibodies (tsAbs)

Many challenges still exist, including dose, treatment resistance, and the first bispecific antibody licensed by the FDA to treat B cell malignancies, blinatumomab. It also has modest efficacy in solid tumors. The development of multi-specific antibodies has received a great deal of attention to circumvent these constraints, providing new opportunities to address the intricate biology of cancer and the beginning of anti-tumoral immune responses. Targeting two tumor-associated antigens at the same time is thought to improve cancer cell selectivity and decrease immune escape. T cell exhaustion may be reversed by co-engaging CD3, and co-stimulatory molecule agonists or co-inhibitory immune checkpoint receptor antagonists in a single molecule [181].

Similarly, NK cells may become more potent killers if two activating receptors are targeted. These are but a few instances of how antibody-based molecules may interact with three or more pertinent targets. Since a single therapeutic drug may yield a similar (or better) therapeutic impact than a mixture of distinct monoclonal antibodies, multi-specific antibodies are attractive from the standpoint of healthcare expenses. Multi-specific antibodies have exceptional qualities despite production difficulties, which could make them more effective biologics for cancer treatment [182].

#### Catalytic antibodies (cAbs)

Research on catalysts had previously been done, but the development of new catalysts was made possible by catalytic antibodies (antibody enzyme). After decades of research, scientists have found many techniques to produce antibodies with specific properties and catalytic capabilities, as well as natural antibodies that can hydrolyze substrates such as proteins, nucleic acids, and polysaccharides. In the fields of biology, medicine, and chemistry, these antibodies are widely utilized. In the area of infection and immunity, where the onset and development of autoimmune illnesses frequently take a long period, catalytic antibodies can still be involved in the process and even completely prevent it [183].

#### Single-chain fragment variable (scfv) Abs

Recombinant full-length mAbs often face limitations in treating human diseases. To address these challenges, smaller-sized scFvs were developed, consisting of VH and VL regions, connected by a flexible polylinker (15–20 aa) [184, 185]. The bacterial expression system, particularly *E. coli*, is a popular method for producing scFvs, allowing optimal folding, improvements in disulfide bond modification, and protein folding, which enhance scFv functionality and stability, making it cost-effective [185]. scFv Abs offers several advantages, including enhanced tissue penetration, rapid blood clearance, reduced renal absorption, and the absence of potentially problematic Fc-mediated immune responses, making them valuable tools for pharmacokinetics, drug administration, and immunogenicity mitigation [186]. Due to recent advancements in library design, construction methods, and target selection, high-affinity scFv can be used as mAb substitutes for the treatment of inflammatory, autoimmune, and chronic viral disorders, including cancer and neurologic therapy [187].

However, the compact size of scFv Abs leads to a shorter serum half-life and potential immunogenicity and susceptibility to aggregation. Additionally, scFvs lack multivalency (limited epitopes) and effector functions associated with the Fc region [188]. Despite these drawbacks, scFv Abs possess significant potential, with ongoing research set to overcome limitations and expand their applications for therapeutic and diagnostic uses. Their unique attributes render them valuable for noninvasive in vivo imaging and treating various diseases. Advancements in stability, multivalency strategies, and production techniques will enhance their effectiveness. Their role in advancing personalized medicine is expected (by rapid production of patient-specific Abs), including combination therapies and novel drug delivery systems [189].

#### Nanobodies (Nbs)

The isolation and successful expression of VHH domains, also known as single-domain Abs or Nbs, were made possible by the discovery of H chain-only Abs in *Trypanosoma evansi*-infected camels. These Abs lacked L chains and CH1 domains and consisted of just two H chains, each with a single variable Ag-binding domain (VHH domain) [190]. Nbs can be obtained from a variety of synthetic, naïve, or immune libraries. Camels are immunized as part of the immune library process, after which Nb is isolated, expressed in *E. coli* systems, and selected using surface plasmon resonance and phage display [191]. These days, VHHs are applied to intrabodies (Abs for intracellular application), cancer, CNS disorders, infectious diseases, and bispecific and chimeric Ag receptor T cell treatment. Caplacizumab, the first treatment based on VHH, has been approved; 16 more are undergoing clinical trials [192]. Nbs, which are smaller than human Abs and may be used as potential treatments, are also produced by sharks, llamas, dromedaries, and alpacas in addition to camels [192].

Because of their unusual structural properties, Nbs have many advantages over ordinary mAbs. Their broad CDR3 domains, convex shape, and small size allow them to bind to Ag sites that are inaccessible to bigger mAbs [193]. Strong binding affinities, remarkable stability, protease resistance, and solubility of Nbs allow for enhanced tissue penetration and blood–brain barrier bridging. Nbs's monovalent format frequently needs to be changed to achieve the required therapeutic and diagnostic capabilities. Their tiny size (15 kDa) allows for quick renal clearance, which limits serum persistence and diagnostic uses. This can be overcome by conjugating the compound with either albumin or polyethylene glycol. Fc region conjugation can alleviate the effector activities hindered by the absence of an Fc region. Additionally, Nbs might not be able to recognize all epitope types, and humanization methods for therapeutic Nbs might affect affinity and refolding [192]. The approval of Nb-based therapies for cancer and autoimmune diseases has sparked a lot of interest in their application in therapeutics and diagnostics.

#### Fc-engineered Abs

All the vital functions of Abs are included in the Fc region of IgG Abs. IgG effector activities, aside from neutralization, depend on the Fc region's interaction with complement component C1q or Fcγ receptors [194]. The immune system and treatment approaches rely on the actions of Fc-mediated Ab. While inhibitory FcγRIIB controls the immune response, activating receptors (FcγRI, FcγRIIA, FcγRIIC, and FcγRIIIA) aid in complement-dependent cytotoxicity, Ab-dependent cellular phagocytosis, and ADCC. While enhancing neonatal FcRn interactions prolongs half-life and boosts mucosal transport, decreasing it improves Ab clearance in autoimmune diseases [195]. Through Fc glycoengineering and Fc-based mutations, Ab engineering can improve Ab functions [196]. IgG1's glycosylation site at N297 is crucial because mutations there have a big impact on FcγR interactions.

Improved ADCC results from removing core fucose, and longer half-life and precise adjustment of effector functions can be achieved through glycosylation modifications. Tugging with the interactions between FcγRIIB and FcRn has shown promise in tumor therapy and Ab pharmacokinetics, respectively [197]. Additionally, Fc engineering makes it easier to produce Abs that preserve target binding while minimizing or removing Fc-mediated effector effects [197]. Fc-engineered Abs have disadvantages despite their many benefits. The altered Fc regions might become more immunogenic, which could result in serious responses. The process of Fc engineering can be expensive and complicated. Engineering and customization may inadvertently impact stability, leading to aggregation or changing pharmacokinetics. Furthermore, it can be challenging to forecast the outcome in different clinical situations due to the variable nature of the precise effects of Fc changes [198]. Careful evaluation of these limitations is necessary during the development and clinical deployment of Fc-engineered Abs to guarantee their safety and efficacy.

#### Ab biosimilar

Biological medications known as ab biosimilars resemble licensed therapeutic monoclonals, sometimes referred to as reference or original mAbs, in terms of structure, efficacy, and function. Biosimilar Abs functions as "generic" versions of original mAbs. They are made from distinct clones and manufacturing techniques, which result in changes in glycosylation and microvariations such as charge variants. These variations can impact the quality, safety, and potency of the Ab. Because mAbs are inherently complicated, biosimilar mAbs do not exactly match the original mAb in terms of safety, efficacy, and quality [199]. Over the past ten years, many biosimilar mAbs have been approved by regulatory bodies. Prominent examples of these include bevacizumab, trastuzumab, and infliximab for use in treating cancer [200].

Because originator mAbs can be prohibitively expensive in developing nations, Ab biosimilars provide an affordable alternative, hence boosting accessibility. As opposed to chemical pharmaceuticals, developing Ab biosimilars calls for more rigorous approval procedures since it requires a thorough understanding of biopharmaceuticals, which is a significant challenge, as well as the structure and function of the original product [201]. Large-scale clinical trials are required to address concerns regarding immunogenicity resulting from differences in glycosylation and contaminants introduced during production, which makes the regulatory process resource-intensive [202]. Regulatory bodies have devised all-inclusive protocols to optimize preclinical, clinical, and approval procedures for biosimilars [203]. Because mAb biosimilars have the potential to greatly increase patient access to potentially lifesaving mAb medicines, continuous scientific study in this field will resolve long-standing concerns about their usage and spur further innovation.

#### Ab mimetics

"Synthetic Abs", or "Ab mimetics", are made to mimic the actions of natural Abs. They stand in for Ag-binding portions of complete mAbs, devoid of the Fc region and its complications [204]. Improved stability, economy, and ease of engineering are some of their benefits. Ecallantide, affibodies, adnectins, affimers, aptamers, designed ankyrin repeat proteins (DARPin), and knottin molecules are a few examples of Ab mimetics. Each of these molecules is made with certain characteristics, such as reduced immunogenicity, protease resistance, and pH stability [205]. There are two main techniques for creating Ab mimetics: (1) protein-directed evolution and (2) CDR grafting with FR sequence homology as guidance [206]. While CDR fusion alters the structure of Ab to generate functional mimetics, protein-directed evolution harnesses evolution's force to enhance binding properties.

Phage display and in silico design are also utilized by the Ab mimetic generation to recognize and anticipate molecules (peptides) with certain binding characteristics. They fall into two categories: (1) peptide mimetics (paratope) and (2) single-domain Abs (Nbs). The latter describes short peptide sequences (1–2 kDa) that imitate mAb binding characteristics. Ab mimetics have many benefits over mAbs, including improved stability, standardization, low-cost manufacture, intracellular application, and decreased immunogenicity and toxicity [207]. However, difficulties for therapeutic and diagnostic uses arise from their shorter half-life, worse interactions with immune cells because of their smaller size, and lack of the Fc region. Techniques such tiny albumin-binding domain (ABD) protein binding, PASylation, and PEGylation are being investigated as solutions to this problem [208].

#### Ab-drug conjugates

A novel class of mAbs-based therapies known as Ab-drug conjugates (ADCs) combines the lethal power of small-molecule pharmaceuticals (conjugates) with the specificity of monoclonal antibodies (targeting) [209]. By delivering strong anticancer medicines to tumor cells specifically, they lessen the systemic damage that comes with conventional chemotherapy. Significant advancements have been made since the FDA approved the first ADC, gemtuzumab ozogamicin, in 2000. Three prominent examples are polatuzumab vedotin for large B cell lymphoma, brentuximab vedotin for Hodgkin lymphoma, and ado-trastuzumab emtansine for human epidermal growth factor receptor 2 (HER2)-positive breast cancer [210]. ADCs have the potential to improve the overall antitumor response when combined with other cancer therapies. Targeted delivery and increased efficacy, effective treatment by concentrating the cytotoxic payload on the target, are two of their many noteworthy advantages.

By focusing on certain Ags or "mechanisms of action". It can also be utilized to overcome medication resistance. It also works well on cancers that are resistant to traditional therapy [211]. The creation of ADCs is a difficult procedure that involves a protracted and expensive development process to identify tumor-specific Ags, conjugate cytotoxic payloads, and optimize pharmacokinetics [212]. Moreover, treatment resistance and ADC effectiveness may be hampered by the variability of Ag expression. ADCs may potentially cause damage in non-cancerous tissues due to "off-target toxicity". Fc may cause the immune system to identify components of ADC as foreign, which could result in immunological reactions directed against the therapeutic drug [213]. The development of ADCs includes challenges such as Ag selection and resistance mechanisms, which need to be addressed to overcome these limitations and harness the full potential of ADCs.

## Current therapeutic applications of mAbs in non-communicable diseases

### Cardiovascular diseases

MAbs have gained increasing importance in the treatment of cardiovascular diseases by selectively targeting proteins or receptors implicated in disease progression, offering a more specialized treatment strategy. In the context of cardiovascular diseases, mAbs address diverse aspects such as lipid metabolism regulation, inflammation reduction, and blood clot management. For example, mAbs targeting proprotein convertase subtilisin/kexin type 9 (PCSK9) control cholesterol metabolism, whereas anti-IL-1β mAbs aid in decreasing inflammation and minimizing the risk of recurring events in patients with prior myocardial infarctions. Additionally, certain mAbs target platelet glycoprotein receptors to diminish clot formation in high-risk situations.

Demonstrating the effectiveness against cardiovascular diseases. Anacetrapib serves as a cholesteryl ester transfer protein (CETP) inhibitor and plays a crucial role in successfully boosting high-density lipoprotein cholesterol (HDL-C) concentrations and decreasing low-density lipoprotein cholesterol (LDL-C). The reduction in LDL-C levels can be ascribed to both the hindered cholesteryl ester transfer from HDL to LDL and the potential drop in plasma levels of the PCSK9, a crucial regulator of LDL receptors [214]. Research examining the impact of anacetrapib on ApoB metabolism in mildly hyperlipidemic individuals revealed that it lowers LDL-C concentrations by augmenting the LDL-ApoB FCR, whether as a sole treatment or combined with statins indicating anacetrapib independent of statin, co-treatment has shown remarkable LDL-ApoB clearance.

**Fig. 4:**
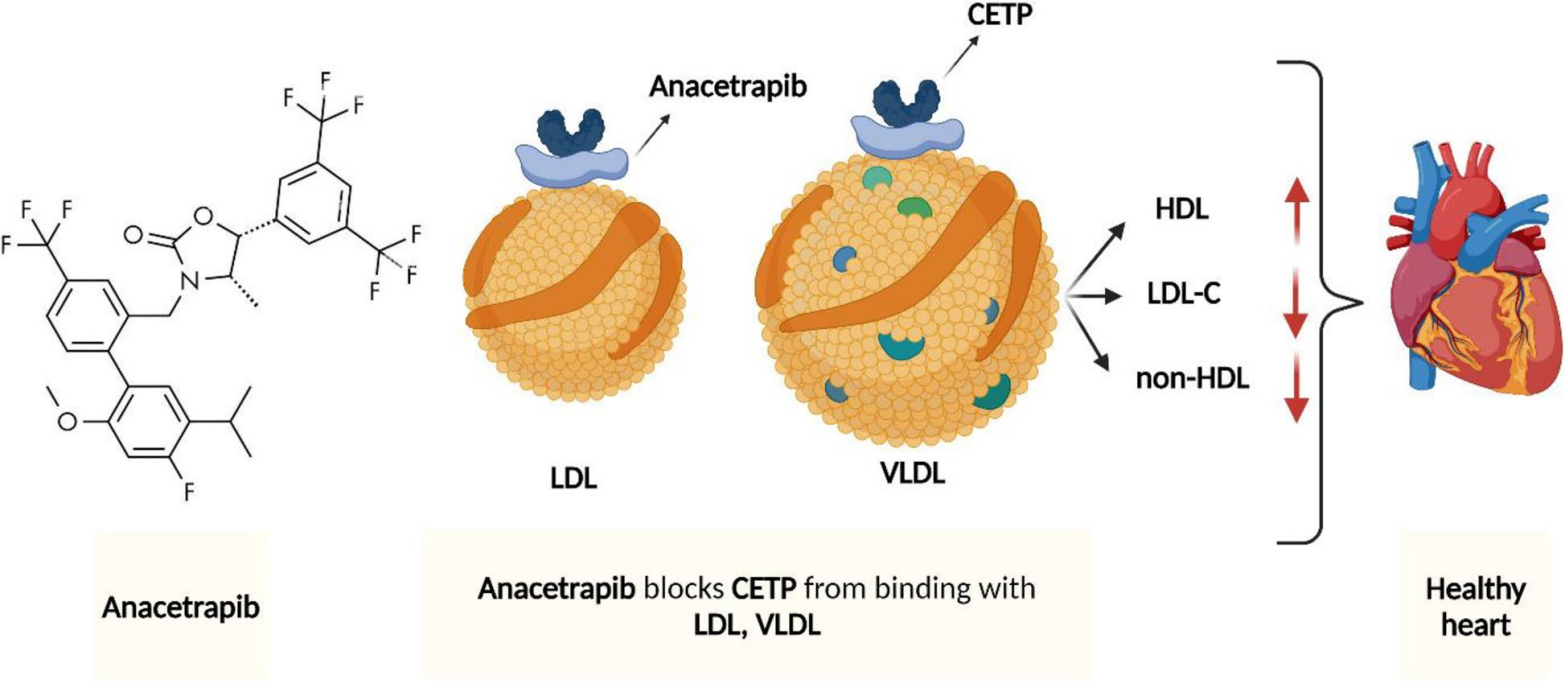
Diagrammatic description of the effect of anacetrapib monoclonal antibody on lipid profile and cardiovascular system

Although encouraging outcomes are presented through randomized evaluation of the effects of anacetrapib through Lipid-modification trials showcasing the ability of anacetrapib to lower the risk of major heart-related events when paired with atorvastatin Fig. 4, the drug has not been approved by the FDA. The primary concern is the lipophilic properties of the drug, which lead to its accumulation in adipose tissue with ongoing use, generating safety concerns during the drug development phase. Consequently, the quest for regulatory approval was discontinued [215]. PCSK9 inhibitors, another example of monoclonal antibodies, have emerged as promising treatment options for reducing LDL-C levels and mitigating the risk of cardiovascular events. These inhibitors specifically target the PCSK9 protein, which plays a pivotal role in the regulation of circulating LDL-C levels. PCSK9 inhibitors such as alirocumab and evolocumab exhibit high specificity for the catalytic domain of the PCSK9 protein.

This binding effectively prevents PCSK9 from interacting with low-density lipoprotein receptors (LDL-Rs) in the hepatocytes, thus averting the internalization and degradation of LDL-Rs within lysosomes. Consequently, LDL-Rs can be preserved and recycled back to the hepatocyte cell surface, augmenting their availability for binding with LDL-C particles and uptake of circulating LDL-C particles. Once internalized, LDL-C particles are broken down inside the hepatocytes, effectively clearing them from the bloodstream [216]. Another example is canakinumab, a human monoclonal antibody that selectively targets and neutralizes the proinflammatory cytokine interleukin-1 beta (IL-1β), which plays a crucial role in various inflammatory diseases such as autoinflammatory disorders, cardiovascular diseases, and some cancers.

The affinity of canakinumab to interleukin-1 beta granted this mAb FDA approval for potent inflammatory conditions including cryopyrin-associated periodic syndromes (CAPS) and systemic juvenile idiopathic arthritis (SJIA) [217]. Canakinumab anti-inflammatory thrombosis outcomes study (CANTOS) trial explored the capability of canakinumab to reduce cardiovascular events in patients with prior heart attacks and high inflammation levels. This study also provided promising initial findings, indicating that canakinumab may contribute to reducing the incidence and mortality of non-small cell lung cancer by affecting the inflammatory pathways related to tumor development [217].

### Respiratory diseases

In respiratory disorders, such as severe asthma and idiopathic pulmonary fibrosis, mAbs selectively target components of the immune system to alleviate symptoms and control disease progression. For example, in asthma, mAbs targeting immunoglobulin E (IgE) or specific interleukins help reduce airway inflammation and improve symptom management. By focusing on these immune system components, mAbs can provide relief to patients who do not respond well to traditional therapies. The targeted action of nintedanib, a tyrosine kinase inhibitor, illustrates the potential for developing mAbs aimed at the pathways involved in fibrotic lung disease progression, such as idiopathic pulmonary fibrosis. Although it is not a mAb, this approach demonstrates the possibility of creating mAbs targeting relevant pathways in respiratory disorders. Mepolizumab and benralizumab are both monoclonal antibodies utilized for treating eosinophilic asthma [218].

**Fig. 5:**
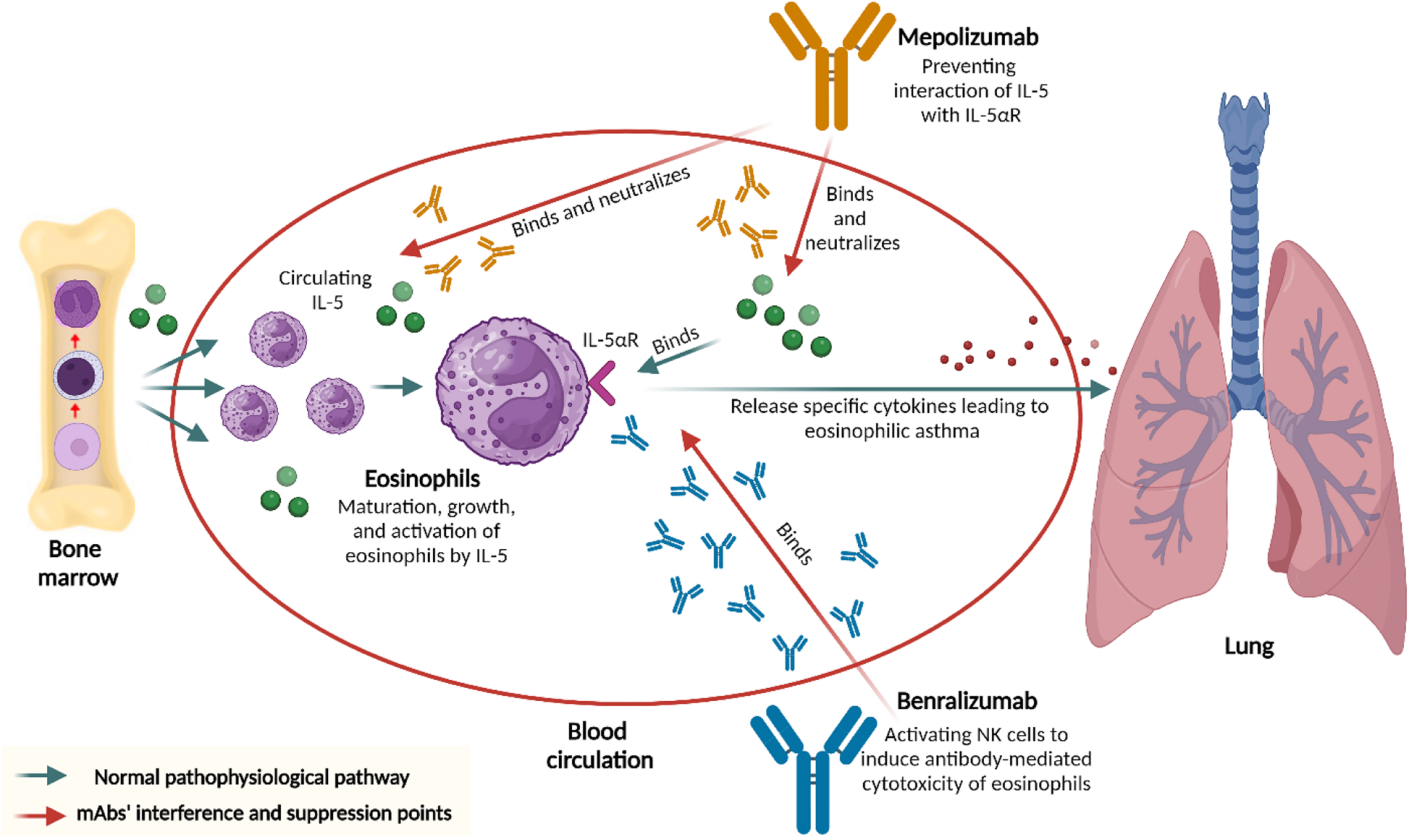
Diagrammatic illustration of the mechanism of two monoclonal antibodies utilized for treating eosinophilic asthma, mepolizumab and benralizumab. Mepolizumab specifically targets interleukin-5 (IL-5), a cytokine crucial for developing, differentiating, and surviving eosinophils in bone marrow. In comparison, benralizumab attaches to interleukin-5 receptor alpha (IL-5Rα) present on eosinophil surfaces

Mepolizumab specifically targets interleukin-5 (IL-5), a cytokine crucial for the development, differentiation, and survival of eosinophils. In comparison, benralizumab attaches to interleukin-5 receptor alpha (IL-5Rα) present on eosinophil surfaces. Mepolizumab is given as a subcutaneous injection every four weeks, taking about four weeks to reduce eosinophil levels in the blood. However, mepolizumab does not eliminate eosinophils and has minimal impact on those in the airway [218]. In contrast, benralizumab is administered subcutaneously every eight weeks and requires only approximately a day to lower blood eosinophil counts. Notably, benralizumab can deplete eosinophils in both the bloodstream and airways [218] Fig. 5. Benralizumab has several benefits over mepolizumab, first, benralizumab is more effective in decreasing eosinophil levels in the airways, second, it can induce eosinophil cell death in tissues, even when other factors favor eosinophil survival, and third, benralizumab has a less frequent administration schedule than that of mepolizumab [219].

Both mepolizumab and benralizumab have received FDA approval for the treatment of eosinophilic asthma. They are effective in alleviating asthma symptoms in eosinophilic asthma patients. Nonetheless, benralizumab may be a better option for individuals with severe asthma or those unresponsive to other treatments because of its more potent mechanism of action and ability to eliminate eosinophils from both the blood and airways. Omalizumab is another monoclonal antibody, effective as an anti-IgE treatment employed to treat nonsteroidal anti-inflammatory drug-aggravated respiratory disease (N-ERD). Its function results in better asthma regulation, decreased exacerbations, and enhanced lung performance in individuals with asthma. Furthermore, it provides positive outcomes for patients with severe chronic rhinosinusitis accompanied by nasal polyps (CRSwNP) and co-occurring asthma [220].

The primary focus of omalizumab is IgE, an essential initiator and mediator of type 2 inflammation, which is distinguished by elevated IgE and eosinophil levels. Omalizumab binds to and neutralizes circulating IgE and, in specific instances, locally produced and tissue-expressed IgE in patients with allergic rhinitis and asthma [220]. A crucial aspect of omalizumab function is the reduction in free IgE levels during treatment. This reduction stabilizes mast cells in individuals with N-ERD, thereby reducing the release of inflammatory agents. Omalizumab prevents the interaction between the high-affinity IgE receptor (FcεRI) and IgE, ultimately leading to decreased mast cell activation. Moreover, omalizumab supports the rapid downregulation of FcεRI expression in immune cells such as basophils, mast cells, and dendritic cells, further mitigating IgE-mediated inflammatory processes.

In an in vitro study conducted by Serrano-Candelas et al. 2017, omalizumab reduced leukotriene production, an inflammatory substance involved in asthma and various inflammatory disorders. Omalizumab manages this by removing prebound IgE from mast cells and basophils, subsequently interfering with the IgE-dependent phosphorylation pathway and ultimately lowering leukotriene generation [220]. Due to the outstanding outcome of omalizumab, the FDA has granted approval for omalizumab to be registered and marketed in the USA for the management of moderate-to-severe persistent allergic asthma and chronic idiopathic urticaria [221]. In contrast to conventional medications, biopharmaceuticals have few side effects and exhibit great targeted specificity and activity rather than symptomatic treatments. MAbs are the most widely utilized biopharmaceuticals for treating a broad spectrum of diseases, particularly autoimmune diseases, coronary artery disease, inflammation, and carcinoma [222, 223].

Given their ability to be tailored to target a specific receptor, monoclonal antibodies, or mAbs, are increasingly being prescribed to patients and investigated in various fields of medicine and research. The first monoclonal antibody was approved by the FDA back in 1986, and ever since then, the engineering of antibodies has undergone a significant evolution, with an increased number of approved monoclonal antibodies which is expected to take control of the biopharmaceutical market [224]. FDA has approved 103 therapeutic antibody drugs since October 2021 [225].

### Autoimmune diseases

More than 100 autoimmune diseases are recognized, among them are systemic lupus erythematosus (SLE), rheumatoid arthritis (RA), Crohn’s disease, and multiple sclerosis (MS) are the most common [226]. MAbs can be used to treat autoimmune diseases by targeting specific molecules involved in the immune response. mAbs used to treat autoimmune diseases usually employ more than one mechanism of action. According to different antigens, the targeted mAbs can directly induce apoptosis of cells or eliminate the antigens indirectly via ADCC [226].

#### Rheumatoid arthritis (RA)

RA is a chronic autoimmune disease known for prolonged joint inflammation, systemic inflammation, and immunological abnormalities [222]. Besides, it creates a significant healthcare burden on people as well as society as the global incidence of RA is around 0.5%-1% [227]. Previously, the only available options for RA treatment were glucocorticoids as well as non-steroidal anti-inflammatory drugs (NSAIDs) [220]. While it has been proven that glucocorticoids provide immediate symptom and disease relief, they are also associated with substantial long-term side effects, on the other side NSAIDs reduce the number of prostaglandins in the body that help with the pain but do not interfere with joint damage and therefore do not cure the disease. However, a significant shift in the RA management landscape was raised with the concept of disease-modifying anti-rheumatic drugs (DMARDs) [227].

They are currently essential in the therapy of RA as they help to coordinate disease progression through anti-inflammatory and immunomodulatory actions. Methotrexate, sulfasalazine, and hydroxychloroquine are the commonly used synthetic DMARDs [223, 227]. When faced with ineffectiveness obtained with the above DMARDs, biological DMARDs (bDMARDs) or targeted DMARDs are used either alone or coupled with one of the conventional DMARDs. bDMARDs are monoclonal antibodies that are genetically engineered to specifically target key inflammatory pathways, molecules and cell mediators of the inflammatory process that cause tissue damage [223, 228]. The classes of bDMARDs currently utilized in RA treatment mainly target tumor necrosis factor-alpha (TNF) inhibition, interleukin-6 (IL-6), CD-20 depletion, and IL1 [228]. Additionally, most of the biological DMARDs as TNF Inhibitors, IL-6 inhibitors, and CD-20 depleting antibodies pose enhanced activity when combined with other conventional synthetic DMARDs [223].

##### TNF inhibitors

Inhibitors of TNF were the first biological DMARDs approved by the FDA to treat Rheumatoid. TNF-alpha is a well-known signaling cytokine important in the pathogenesis of RA, via various mechanisms [229]. Five of the TNF inhibitors are currently approved for use in rheumatoid therapy, infliximab, certolizumab pegol, etanercept, adalimumab, and golimumab. It has been proven from clinical studies that treatment with TNF inhibitors improves the implications of rheumatoid arthritis and has been demonstrated better effectiveness when coupled with methotrexate than when each is given alone [230–233].

##### IL-6 inhibitors

IL-6 is a pivotal cytokine involved in the pathogenesis of RA and autoimmune diseases [234]. Currently, the two FDA-approved mAbs, tocilizumab and sarilumab antagonize the IL-6 receptor by targeting site 1 [235, 236] whereas olokizumab works by binding to site 3 blocking the interaction with gp130 the signaling co-receptor [237]. Olokizumab is used with patients with incomplete response to TNFα and has proved its safety and effectiveness. Head-to-head clinical trials with adalimumab have demonstrated that IL-6 inhibitors have greater efficacy than TNF inhibitors when used as monotherapy without concurrent csDMARD [238, 239].

##### CD-20 depleting antibodies

Rituximab is a monoclonal antibody that binds to the B-cell surface molecule CD20 causing B-cell depletion. B cell therapy in RA aimed to reduce the production of pathogenic autoantibodies. If the patients suffer from incomplete responses to one previous TNF inhibitor, switching to rituximab is a better alternative to another TNF inhibitor [233]. However, some countries restrict its application as a first-line bDMARD and many others require prior failure of a TNF inhibitor before using it.

#### Systemic lupus erythematosus (SLE)

SLE is a challenging heterogeneous autoimmune disorder [240] where the immune system produces autoantibodies, particularly nuclear antigens, which then form immune complexes that lead to tissue deposition and cytokine induction targeting different organs such as kidneys, brain, and heart [240] resulting in chronic inflammation. One of the most common consequences is lupus nephritis, where kidney inflammation hinders the clearance of wastes and toxins. A 4-year survival rate of ~ 50% in 1950 improved to a 15-year survival rate of ~ 85% in 2013. However, patients suffered from atherosclerosis, osteoporosis, increased infection risk, and untimely death. An analysis of patients with lupus nephritis demonstrated that commonly used drugs are unlikely to produce any beneficial effects [241]. Indeed, belimumab, a humanized anti-B cell activating factor (BAFF) monoclonal antibody that prevents BAFF from attaching to its receptors on B cells, is the only licensed biological treatment for SLE [242]. Although belimumab was successful in phase III trials and is approved for the treatment of SLE [243], many patients didn’t respond to this medication, and it is unknown whether it will help with lupus nephritis because those with kidney diseases were not in the trial [241].

### Diabetes

Type 1 diabetes (T1D) is a T-cell organ-specific autoimmune illness that develops when the beta cells secreting insulin in islets of Langerhans selectively malfunction [244, 245] leading to reduced insulin production [246]. External supplementation with insulin is the current standard treatment. Although this therapy has made considerable advances, there are still drawbacks, such as the risk of hypoglycemia and serious degenerative implications [247]. Immunotherapies that specifically restore lasting cell-specific self-tolerance are currently needed for treating T1D, according to [246]. One method for preventing and/or treating T1D has been the use of mAb. In T1D, mAb therapy must stop the continuous loss of beta cells while restoring long-term self-tolerance. Therapeutic mAb often works in two general ways: (1) reducing target cell populations, and (2) blocking cell receptor activity.

Use of monoclonal antibodies that target CD3 is a possible intervention method since CD3 is a molecule associated with T cell receptor (TCR) to recognize antigens. According to [247, 248] CD3 monoclonal antibodies interfere with T cell activation and partially deplete T cells. Preclinical reports suggested that anti-CD3 can reverse Type 1 diabetes by inducting a population of regulatory T cells [230, 249]. It has been reported that a single course of treatment with anti-CD3 mAb, hOKT3gamma1(Ala-Ala), can help preserve insulin production in new-onset type 1 diabetes patients for longer than 1 year after administration [249, 250]. Humanized Fc-mutated CD3 antibodies have been thoroughly explored in clinical trials and have produced the best results. Phase I, II, and III of clinical trials made on two different monoclonal antibodies, teplizumab, and otelixizumab, demonstrated effectiveness in patients with recent onset hyperglycemia [251]. A 14-day treatment [251], which began 4 to 12 weeks after insulin treatment reliably maintained C-peptide levels for 2–4 years in newly diagnosed patients [251, 252]. Oral delivery of CD3-specific antibodies is a successful treatment method for autoimmune disorders. Recently, teplizumab was approved by the FDA as the first preventative medication for T1DM [253, 254].

### Cancer

Immunotherapy using monoclonal antibodies is now considered to be one of the available cancer therapies, besides chemotherapy, surgery, and radiation. MAbs have various modes of action and properties that aid their prevalence. Simultaneously, antibodies can target tumor cells directly and promote the induction of long-lasting immunological responses [255]. In cancer immunotherapy, monoclonal antibodies can exert their effect by direct targeting mediated by the variable portions of the molecule or indirectly by the Fc portion; or even by both mechanisms simultaneously [256].

#### Blocking cell signaling

Cancer development is strongly controlled by many cell-signaling pathways governed by the ligand-receptor interactions. This is evident during angiogenesis, proliferation, growth, and cell survival. Therefore, mAbs are engineered to inhibit cell–cell signaling, via binding to growth factors, cytokines, or receptors [257]. Commonly available antibodies include bevacizumab, a humanized IgG1, against vascular endothelial growth factor A (VEGF-A) [257]. Also, cetuximab an anti-EGFR mAb induces apoptosis [258, 259].

#### Antibody-dependent cellular cytotoxicity (ADCC)

In ADCC, the mAb works to connect between the cancer cell and the immune system effector cells (NK cells, dendritic cells, and macrophages). With the cancer cells, the mAb starts an antigen-specific binding via Fab fragment, while, with the immune cells, it interacts through the Fc domain and cell surface receptors [257]. Trastuzumab and pertuzumab are monoclonal antibodies for HER2, both are mAbs that exert direct mechanisms of action on the antigen, simultaneously generating ADCC as proved by [257]. Rituximab, an approved anti-CD20 mAb induces ADCC by binding to CD20 [260, 261].

#### Antibody-mediated complement-dependent cytotoxicity (CDC)

For mAbs to communicate through interacting with a set of plasma proteins, they must engage in complement-dependent cytotoxicity. The complement system is made up of a series of dormant enzymes that, when they are activated, help lyse the target cell by starting a proteolytic cascade [262]. The complement system starts when mAbs attach to the tumor cell and the C1 protein complex binds to the antibody's Fc domains. Moreover, each of the remaining proteins in the system is drawn in and split into two pieces one by one. While one of the two fragments is covalently linked to the target cell's membrane and fused to the resulting proteolytic complex, the other fragment returns to circulation to carry out biological duties. The process culminates in the formation of a membrane attack complex (MAC), which permits pores to open on the surface of tumor cells, causing cell rupture and death [262]. Different mAbs can generate CDC, two of which are human IgG1 ofatumumab and humanized IgG1 alemtuzumab [263].

#### Activation of immunological checkpoints

In Cancer immunotherapy, mAbs can be used as checkpoint inhibitors for example programmed cell death 1 ligand 1 (PD-L1) antibody and cytotoxic T lymphocyte-associated antigen 4 (CTLA-4). After repeated antigen interactions in the body, T-cells start to express inhibitory checkpoints to stop further activation and tissue damage. Both CTLA-4 and PD-1 are part of the inhibitory receptors important for ensuring T-cell tolerance via blocking this antigen interaction, through binding to either the inhibitory checkpoint or to its ligand, therefore stopping the suppression of T lymphocytes [264, 265]. Ipilimumab is, till now, the only FDA-approved anti-CTLA-4 antibody [266]. Pembrolizumab, nivolumab, atezolizumab, and durvalumab are approved by the FDA for blocking the PD-1 signaled pathway [267].

## Ongoing development of mAbs-dependent antimicrobials for communicable diseases

### Viral diseases

When applying antibody treatments, viruses are classified according to whether they have an envelope or not. The viral glycoproteins of the envelope on the surface of the virion are target antigens that antibodies recognize. The glycoprotein recognizes and communicates with a host cell receptor through a binding site on the glycoprotein [268–270]. When the viral envelope fuses with the host's cellular membrane, the viral capsid and genome can enter the host. Among the viruses that are enveloped include SARS-CoV, SARS-CoV-2 [271–288], influenza viruses [289–302], EBOV [303–309], BDBV [306–308], SUDV [309], HCV [310], HBV [311], ZIKV [312–318], DENV [318, 319] CHIKV [320, 321], and HIV [322–325]. Neutralizing mAbs against enveloped viruses is primarily intended to stop the glycoprotein from binding to the receptor on the host cell. However, non-enveloped viruses, like adenovirus, norovirus, and rhinovirus, enter the host by lysing the membrane or forming structures resembling pores in the membrane, all without requiring a host receptor to attach themselves to the viral glycoprotein. There hasn't been much development of antibodies against non-enveloped viruses [268, 269].

### Bacterial diseases

#### Direct approach (antibacterial antibodies)

Numerous components and virulence mechanisms are involved in bacterial pathogenesis, and mAbs have demonstrated the ability to act across multiple pathogenesis phases. The mAbs can bind molecules that have been released, such as toxins or quorum-sensing signaling molecules [326], as well as proteins and exopolysaccharides found on cell surfaces, as well as the polysaccharide structure of capsulated bacteria [327]. The outer membrane vesicles, capsular polysaccharide, and lipopolysaccharide (LPS) are among the structures that can be targeted. Gram-negative bacteria mostly comprise LPS, which comprises lipid A, an O-specific oligosaccharide chain, and a core oligosaccharide. LPS of *Legionella pneumophila (L. pneumophila)* is chosen as the target structure against which antibodies were produced as LPS is an immunogenic molecule capable of activating Toll-like receptor 4 causing inflammation [328, 329].

The effectiveness of two mAbs was examined by Cohen et al*.*2017, who worked on anti- *Klebsiella pnemoniae* (*K. pneumoniae*) LPS-O-antigen mAbs. Both mAbs could improve neutrophil-mediated opsonophagocytic killing, although they had different neutralizing activities. They demonstrated that in mice infection models, LPS neutralization greatly decreased mAb protection, and the primary mechanism of action was proven to be opsonophagocytic death. Consequently, it is essential to determine whether mAbs work in concert with the host immune system to achieve their primary protective function when considering LPS as a possible target for mAbs [330]. The bacterial cell wall is encircled by densely packed repeating polysaccharide units that make up the capsular polysaccharide. It would be relevant for mAbs to target this structure because it is linked to resistance to antimicrobial drugs and defense against host immune responses [331].

Antibodies against the capsular polysaccharide are thought to offer a significant degree of protection against *Streptococcus pneumoniae* (*S. pneumoniae*) [332]. Gram-negative bacteria emit outer membrane vesicles, which are nanostructures derived from the outer membrane. Because they include soluble periplasmic material and are made of proteins, lipids, and glycans, they are a valuable tool for the finding of monoclonal antibodies [333]. Because outer membrane vesicles are immunogenic, nonreplicating copies of their parent bacteria and can be used to measure monoclonal antibody binding without cytoplasmic protein interference, they have been employed as a vaccination platform for *Neisseria meningitidis* (*N. meningitidis*) [334]. Among their primary modes of action are complement-dependent cytotoxicity (CDC), antibody-dependent cellular phagocytosis (ADCP), ADCC, and neutralization and inhibition of adhesion which are mediated by Fab antibody structure. Every mAb approved by the FDA targets endotoxins by neutralization.

For bacterial pathogens to successfully infect host cells, they must first adhere to the cells by binding specific receptors on their surface with adhesins, then colonize and invade the cells [335]. When *Bordetella pertussis* (*B. pertussis*) initially interacts with epithelial cells, it is shown that the bacterial proliferation is accelerated [336]. It has been observed that cells produce growth-promoting biological substances and trigger bacterial replication signaling by interacting with bacterial adhesins. Thus, antibodies that disrupt this process can prevent the infection from spreading and prevent the creation of conditions that encourage the growth of bacteria. It has been demonstrated that antibodies targeting filamentous hemagglutinin (FHA) and fimbriae (FIM) 2 and 3 of *B. pertussis* can prevent the bacterium from adhering to epithelial cells in vitro [336, 337].

Additionally, mAbs can opsonize pathogens, enabling phagocytosis in phagocytic cells including neutrophils, macrophages, and monocytes. Since opsonophagocytic activity is regarded as a key indicator of antibody protective efficacy, it can be very important [327]. Certain bacteria, on the other hand, have evolved intracellular survival strategies that allow them to either avoid the lysosome or prevent it from forming [338]. However, mAbs may also promote the release of neutrophil extracellular traps (NETs) or limit the bacterium's ability to survive in macrophages by enhancing phagosome maturation [339, 340].

It was demonstrated that the primary mechanisms of action of two anti-capsular polysaccharide mAbs produced against hypervirulent (hv) *K. pneumoniae* invasive infections were FcR-mediated phagocytosis and enhanced release of NETs. Their efficacy in providing protection was also verified in vivo, as they demonstrated it in several mice models. They inhibit the spreading of hv *K. pneumoniae* from the stomach to other organs in colonized mice [327]. Antibodies can activate the complement cascade in addition to ADCP. This aids in pathogen removal. The quorum-sensing system and biofilm development are two other potential inhibitory functions of mAbs that have been studied in vitro. Bacterial populations known as biofilms play a significant role in the survival of bacterial infections [341], which can shield bacteria from human protection [342] and antibiotic therapy [343]. Membrane-bound protein and carbohydrate components function as adhesins that facilitate cellular adhesion to abiotic surfaces, hence mediating biofilm formation [344].

Antibodies directed against those molecules may interfere with the production of biofilms by causing disruptions in cell-surface and cell-to-cell contacts [345]. Three mAbs that inhibited the formation of biofilms on abiotic surfaces were discovered against *Staphylococcus epidermidis* (*S. epidermidis*)’ cell-wall-bound accumulation-associated protein (AAP). These mAbs decreased *S. epidermidis* biofilm production by as much as 66% when used alone, and as much as 87% and 79% when used in conjunction with other mAbs [345].

**Fig. 6:**
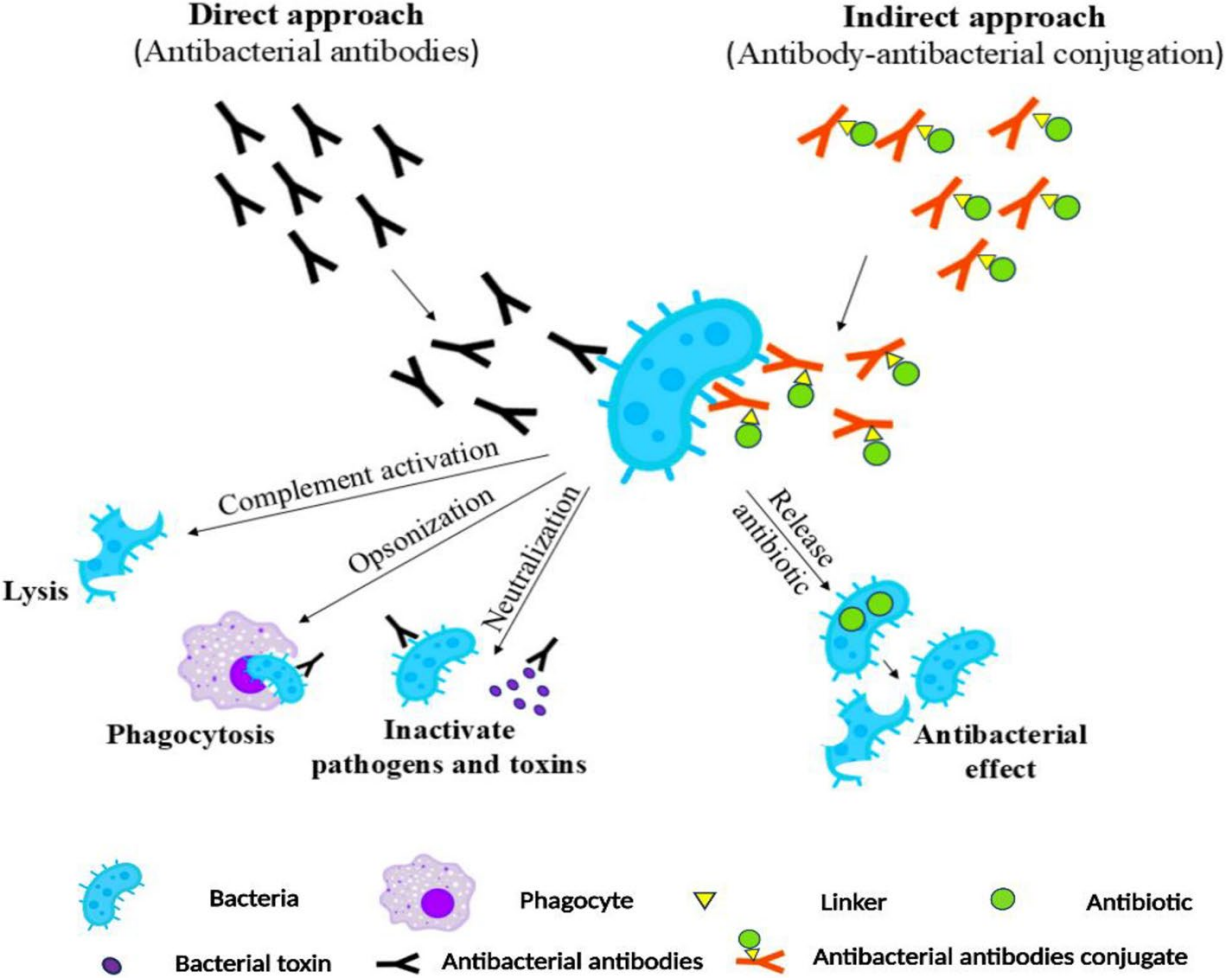
Illustrative diagram of the two main approaches of the antibacterial therapeutic antibodies. In the direct approach, three possible modes of action can occur; inactivate pathogens and toxins by neutralization, phagocytosis of the bacteria through opsonization, and lysis of the target bacteria by complement activation. In the indirect approach, antibiotics bind to antibodies by a linker, the antibody attaches specifically to its target receptor on the surface of the bacteria where the antibiotic is released, performing its destructive action on the target bacteria

A different team discovered a few human mAbs that can recognize both in vivo and in vitro biofilms of *Staphylococcus aureus* (*S. aureus*). The mAbs were divided into two classes: one that recognizes *S. aureus* in both biofilm and planktonic state (i.e., free-floating bacteria) and the other that binds *S. aureus* exclusively in biofilm state. It is possible to target *S. aureus *in vivo over the whole infection cycle by using mAbs that target bacteria in both phases [346]. When the biofilm is broken, scattered bacteria would revert to a more drug-sensitive planktonic condition and be more vulnerable to given antibiotics if mAbs can function in concert with antibiotics [347] Fig. 6.

According to [348], a mAb directed against a specific portion of the *S. aureus* biofilm matrix can break biofilm. Finally, quorum sensing interference has been investigated as a potential strategy to prevent the production of biofilms [347]. Bacteria use chemical signaling molecules called autoinducers to communicate in response to changes in cell population density. These chemicals control gene expression and impact virulence activities [349]. The highly conserved N-acyl homoserine lactones (AHLs), which are produced extracellularly, are an example of a type of signaling molecule [350]. 3-oxo-C12-HSL, an AHL molecule that *Pseudomonas aeruginosa* (*P. aeruginosa*) utilizes, has a regulatory function that affects the production of virulence factors and the creation of biofilms.

#### Indirect approach (antibody-antibacterial conjugates)

Currently, over 100 ADCs are being evaluated globally, mostly for the treatment of cancer [351, 352]. The development of ADCs is difficult, despite the relative simplicity of this antibody-based molecular platform. Since mouse domains were employed to design antibodies, early ADCs showed significant immunogenicity, limited potency, and unsatisfactory target selectivity [353]. However, new approaches to humanization are helping to create next-generation ADCs that are less immunogenic, more powerful, and more selective. The application of ADCs for cancer has taught us lessons about infectious illnesses. Naturally, very strong antibiotics are coupled to mAbs to create AACs rather than a cytostatic medication [354]. An effective antibiotic, a stable cleavable or non-cleavable chemical linker, and a bacterial antigen-specific mAb are required in an AAC for infectious disorders.

Targeting the antibiotic specifically and delivering it straight to the infected location is the main job of the mAb [355]. Therefore, it's crucial to choose the target antigen that the AAC binds to. To minimize off-target effects, the ideal target antigen should be nearly nonexistent in healthy tissues and uniformly expressed on the surface of targeted bacteria [356]. High-affinity binding of the mAb is necessary for both long-term retention and selective accumulation. Furthermore, for the antibiotic to be delivered intracellularly, the antibody-antigen complex needs to internalize because of antibody binding [357]. Because of their great abundance and importance in the pathophysiology of bacteria, carbohydrates are thought to be a possible target for antibodies [358]. LPS is considered one of the antibiotics targets one of the most effective tactics has been to focus on a few elements within this class [359, 360].

Among the promising targets for immunotherapy are highly conserved exopolysaccharides, pilus formation proteins, and extracellular vesicle components [361]. In addition to target selection, which is crucial, consideration should also be given to the IgG type of immunoglobulin as these can affect different AAC effector activities [362]. IgG1 which is one of the longest half-lives can robustly enhance CDC and ADCC [353]. These characteristics may provide an extra therapeutic advantage, even though AACs do not require the antibody to have any further activity beyond binding [354].

Typically to achieve a promising and efficient ADC, that is formed by an antibody targeting a tumor-specific antigen or a related antigen and several payloads through appropriate linkers, three structural points should be carefully studied, chosen, and designed; (1) antibiotic payload(s) which will have the expected and desired effect based on its mode of action, (2) linkers which properly and efficiently link the mAb with the payload, and (3) site-specific conjugation which refers to the process of attaching drug molecules to specific sites on antibody molecules to ensure that the drug attaches to the designated location on the antibody, thereby minimizing the chance of random or nonspecific binding [351–362].

##### Antibiotic payload

Many antibiotics have been studied concerning treating infectious illnesses. However, the bioavailability, toxicity, and biodistribution of these substances can occasionally hinder their clinical utilization [363]. Due to these disadvantages, as well as their short half-life and short-acting action, increasing numbers of high doses are needed in therapy regimens. An innovative workaround for these drawbacks is conjugation to mAbs [281, 364]. The primary mechanism of action of AACs is antibiotics. These antibiotics should possess, among other crucial characteristics, the following: (1) bactericidal potency in the sub-nanomolar range, (2) a functional group for conjugation to mAbs, and (3) solubility and stability in physiological circumstances [364]. The most effective class of antibiotics conjugated to mAbs thus far has been rifamycin-type antibiotics [354].

But various antibiotic moieties, including ampicillin, vancomycin, imipenem, doripenem, gemcitabine, dalbavancin, sitafloxacin, teicoplanin, triclosan, naphthyridine, radezolid, azithromycin, novobiocin, retapamulin, daptomycin, GSK-2140944, CG-400549, and ampicillin, were also successfully conjugated to mAbs and are being researched to combat infectious diseases [365].

##### Linker

The engineering of a linker, which connects cargo and payload, such as an antibiotic and an antibody, is a crucial structural element of an AAC. To maintain the antibiotic's attachment to the antibody, the linker needs to be stable while the blood is flowing [366]. Antibiotics that are circulating too soon can reduce the effectiveness of AAC and increase its toxicity [367, 368]. But, after the antibody is internalized, it still needs to be able to release the payload. The linker hydrophobicity is another characteristic to consider [369]. A hydrophobic payload coupled with a hydrophobic linker may encourage aggregation, jeopardizing the stability of AAC. Because of their utility, several tactics have been extensively used to enhance their physicochemical characteristics. Cleavable linkers and non-cleavable linkers are the two main classes now [370]. Most ADCs in preclinical and clinical research are made up of cleavable linkers. These linkers contain motifs that can release the medication from the antibiotic carrier in response to physiological cues like low pH or proteolytic cleavage [371].

Using established pharmacokinetic (PK) and pharmacodynamic (PD) characteristics of the free payload, this method enables researchers to calculate the potency of the unconjugated payload. Because the hydrazone linker is acid-cleavable, the conjugate can be stable in circulation at a pH of neutral [370]. However, it releases free medicines by hydrolysis in the acidic cellular compartment, either in the lysosomes (pH approximately 4.8) or the acidic endosomes (pH 5.0–6.0). Thus far, in clinical trials, ADCs designed with these linkers have been linked to non-specific drug release because hydrazone linkers hydrolyze slowly at physiological pH 7.4 and 37 °C. The cathepsin B-responsive linker is another significant linker that is utilized in the AAC that is being evaluated clinically [372]. Many cancer cells and bacterial infections overexpress the lysosomal protease cathepsin B [373].

Certain sequences, such as valine-citrulline (Val-Cit) and phenylalanine-lysine (Phe-Lys), are preferentially cleaved by it. The antibiotic is delivered in a traceless manner within the lysosomes upon internalization of the AAC. Among the most effective cleavable linkers for ADCs has been this one. The glutathione-sensitive linker is another cleavable linker that shows promise. This tactic makes use of the increased glutathione concentration ratio that exists between the external environment and the cytoplasm [374]. The disulfide bond is quite stable when it is in circulation. High glutathione levels, on the other hand, cleave the disulfide link during internalization and release the free payload [375]. Compared to conventional linkers, pyrophosphate diester linkers have shown improved circulatory stability and water solubility.

The payload was released by the pyrophosphate diester's rapid cleavage of the linker via the endosomal-lysosomal pathway after internalization [376]. Lastly, there is also the application of a quaternary ammonium salt linker. This tactic was developed to capitalize on a recently discovered link to tertiary amines [377]. Many antibiotics and anticancer medications frequently contain these tertiary amines [371, 378]. To attach the linker, the current method depends on removing a methyl group, which could have an impact on the drug's stability and effectiveness. This novel approach made it possible to create strong ADCs and AACs with more conjugate stability. Compared to cleavable linkers, non-cleavable linkers, which frequently have a thioether bond, are more stable because they can withstand proteolytic destruction. After internalization, they rely on lysosomal breakdown to release the payload [379]. The stability of the payload determines how well this linker works. The medication must continue working even after it connects to the linker [380]. Because of their increased plasma stability, these ADCs are thought to have a better therapeutic index [381].

##### Site-specific conjugation

For the designed AAC to be effective and non-toxic, careful consideration must be given to the choice of antibody, antibiotic, and linker [382]. An additional crucial aspect to consider when choosing the method used in the conjugation of the antibiotic and antibody. The two most significant techniques now in use are enzymatic and chemical conjugation [370]. Researchers can attach an antibiotic to an antibody using a variety of chemical conjugation techniques [383]. The fundamental idea involves a regulated reaction between the antibody's surface-accessible amino acid residues and the reaction handle attached to the linker. A combination of AAC species with different drug-antibody ratios (DARs) and tethering sites can be obtained, depending on the technique chosen [370]. This heterogeneity could jeopardize the AAC's stability, safety, and effectiveness. Therefore, it's critical to choose the best approach for each complex system. The lysine amide coupling method is the most used conjugation technique.

On the surfaces of antibodies, lysines are often exposed and available for conjugation to an activated carboxylic acid group. Nonetheless, antibodies have a considerable degree of variability due to their approximately 80 lysine amino acid residues [384]. This heterogeneity is related to conjugation locations as well as distinct DARs. By altering the drug and antibody stoichiometry employed in the reaction, the former can be reduced to a minimum. For the latter, a few reactive groups must be blocked. Furthermore, the significance of certain lysine amino acid residues in the antibody-antigen interaction may also impair the antibody's ability to bind [385]. All previous trials concluded that the lysine-based approach needs fine-tuning optimization to overcome the significant heterogeneity.

An alternate approach is cysteine coupling, which involves the formation of a disulfide link between the antibody's cysteine amino acid residues and a thiol-reactive group on the antibiotic [386, 387]. However, because cysteine amino acid residues are very reactive, almost all of them form disulfide bonds, meaning that antibodies do not include free thiols. For example, there are four interchain and twelve intrachain disulfide linkages in IgG1, the most prevalent IgG subtype utilized in the engineering of ADCs [370]. The stability of the antibody does not depend on the former. Therefore, in mild circumstances, they can be reduced to produce 2, 4, 6, or 8 free thiols. The twelve intrachain disulfides usually stay whole. Because there are fewer conjugation sites in this method than in the lysine amide coupling, more homogenous ADCs are created. The addition of two additional cysteine amino acid residues for selective antibody binding improves this strategy [388].

Using thiomab, an engineered cysteine technique, extremely homogeneous AACs with a DAR of 2 can be produced. The disulfide re-bridging method is an additional intriguing tactic. In theory, this approach generates conjugation points particular to the site, enabling structural stability, homogeneity, and DARs of 4 [389]. The creation of homogeneous conjugates is another benefit of introducing whole domains or proteins into antibodies. The primary technique in this category is expressed protein ligation (EPL), which forms a new amide bond with the therapeutic payload by activating the target protein's C-terminal using self-splicing inteins (intervening proteins) [390]. Finally, there is increased interest in non-natural amino acid engineering. A possible tactic to carefully manage DARs is to introduce non-natural amino acid residues at certain antibody sites [390].

The non-natural amino acid residues acetylphenylalanine, p-azidomethyl-L-phenylalanine [391], and N6-((2-azidoethoxy) carbonyl)-L-lysine [392] have all been employed by researchers. While the utilization of non-natural amino acid residues permits a high degree of homogeneity, this approach necessitates specific methods and biological agents for the genetic engineering process, which may elicit unfavorable immune reactions [370]. It has been suggested that a few enzymes be used to conjugate medications to antibodies. These enzymes' specificity alters the antibody in a way that is site- or amino acid sequence-specific. Therefore, site-specific conjugation resulting in finely regulated DARs is often possible using enzymatic methods [353].

Three primary conjugation techniques rely on enzymes. Sortase is used in the first step of transpeptidation. The *S. aureus* enzyme sortase A can identify the Leu-Pro-x-Thr-Gly (LPXTG; X: any amino acid residue) pattern [393]. This enzyme connects an olygoglycine-containing molecule by cleaving the Thr-Gly link. Many effective examples of conjugating peptides, proteins, nucleic acids, and other compounds have been reported in the literature [394]. The utilization of microbial transglutaminase for transpeptidation is implied by the second methodology. Drug payloads have been successfully incorporated into antibodies at specified sites with the use of transglutaminases [395]. These enzymes catalyze the process of transpeptidation, which involves covalently coupling a primary amine-containing linker to the primary amine side chain of a particular Glu (Q295) in antibodies.

The final product has a DAR of 2, which is defined. Compared to the other conjugation approaches, the lack of genetic engineering is favorable [396]. Lastly, the engineering of N-Glycan. A conserved Asn (N297) in the Fc domain and the N-glycan on this residue are shared by all IgG classes. It is therefore highly appealing to use this site-specific point to create homogenous conjugations. One method that researchers employ is the addition of an aldehyde group to the N-glycan terminal [397]. Subsequently, these groups can be combined to create pharmacological payloads with aminooxy functions. However, there may be some variation seen. An alternative strategy is to add artificial saccharides to the antibody that have an orthogonal reaction handle. The reproducibility of this method within conjugations is its greatest advantage.

Because they have the potential to impact the overall effectiveness of AACs, these various components must be chosen carefully. Furthermore, it is imperative to consider the PK and PD of the conjugates, as each element is crucial and may have varying effects on the conjugate's effectiveness. Therefore, a thorough PK/PD assessment is essential to verify a particular AAC.

### Fungal diseases

Fungal pathogens represent a major threat to immunocompromised individuals [398, 399]. Mortality rates associated with deep mycoses are generally high, reflecting shortcomings in diagnostics as well as limited and often insufficient treatment options. Apart from the development of novel antifungal agents, it is a promising approach to activate antimicrobial mechanisms employed by the immune system to eliminate microbial intruders. Antibodies represent a major tool to mark and combat microbes. Moreover, mAbs are highly specific reagents that open new avenues for the treatment of fungal diseases. However, studies in which mAbs have been used to combat experimental fungal infections are limited to a few pathogenic yeasts; *Cryptococcus* [400–422] and *Candida* [423–454], dimorphic fungi; *Histoplasma* [455–458], *Paracoccidioides* [459–463], and *Sporothrix* [464–466], and molds; *Aspergillus* [467–484], *Rhizopus* [485], and *Scedosporium* [485].

### Parasitic diseases

Two approaches can be used for the production and application of mAbs in the setting of protozoan infections. The first involves using antibodies to target immunological factors and other host antigens. Using this tactic, one can modulate host immunity to prevent inflammation-induced harm or to create a more effective response for the removal of parasites. The three main benefits of this kind of approach are as follows: (1) the potential to use previously developed, tested in clinical trials, and approved drugs through the process of drug repurposing, (2) the prevention of resistance development and antigenic variability undermining therapeutic efficacy, and (3) the potential for special applications in the context of chronic infections where the pathology is exacerbated by the host response.

However, this tactic necessitates a thorough understanding of the immunomodulation and host–pathogen interaction processes, which are yet mostly unknown [486].

As an alternative, mAbs that specifically target parasitic antigens can be used to cause the elimination of parasites by a variety of mechanisms, such as complement-dependent cytotoxicity, antibody-dependent cellular phagocytosis, and cellular cytotoxicity [487]. However, because most protozoa exhibit antigenic diversity, and because strains differ from one another, identifying the pertinent highly conserved targets to produce such mAbs might be challenging. Furthermore, the success of this tactic hinges on having a thorough understanding of the metabolic pathways, adaptive mechanisms, and parasite life cycle, all of which are too frequently lacking. Therefore, up till now, most antiparasitic mAbs developed or under developmental trials are directed toward a small number of parasitic agents; *Echinococcus* [488], *Leishmania* [489–511], *Trypanosoma* [512–533], *Malaria* [534–550], *Toxoplasma* [551–570], *Trichomonas* [571–573], and *Cryptosporidium* [574].

## Challenges, gaps, limitations, and possible adverse reactions associated with the use of mAbs

Enhancing mAb formulations in terms of dose, distribution, and stability is a continuous challenge. Improvement of these Abs' PK and PD is also necessary. It's important to comprehend how mAbs work in concert with other medications or therapies and the best ABD combinations for different medical conditions. The mechanisms of action of mAbs require further research, particularly in complex illness settings. The development of AI- and machine learning (ML)-based models that can be utilized to further optimize the molecular architectures of protective Ab molecules is hindered by the scarcity of experimentally confirmed data. More sophisticated machine learning models and algorithms must be developed to improve prediction. Next-generation mAb production is expensive and time-consuming. It demands appropriate, scalable, cost-effective cell line production and procedures, as well as efficient expression and purification platforms [575–585].

To reduce any possible adverse effects and immune response, research on immunogenicity risk analysis and long-term impacts related to next-generation mAbs is required. There is a need for more effective techniques to display a wider variety of Ab forms, like multi-specific Abs, Ab fragments, or bsAbs that can target many disease pathways at once. It is necessary to enhance the effectiveness, specificity, half-life, and cost of both traditional and next-generation Abs. It is necessary to create strategies for customizing mAb therapy for individual patients according to their clinical, immunological, and genetic factors. To increase the range of diseases that can be treated with Abs, new delivery techniques are required, such as oral or inhalation administration. Future research should be focused on actively investigating ADE mechanisms to minimize potential risks in case of therapeutic mAb development [585].

As previously demonstrated, mAbs are proteins genetically modified to resemble the structure of naturally occurring human proteins. As such, they are processed differently by the body than pharmaceuticals. Five types comprise the adverse drug reactions (ADRs) linked to the use of monoclonal antibodies [575–579]. "Cytokine syndrome reactions" are types of alpha reactions, defined as excessive cytokine production and release in the systemic circulation. Allergy-like responses known as type beta reactions can be mediated by T cells, IgE, or IgG (immediate vs. delayed). Autoimmunity, atopic or allergy diseases, and immunological dysfunction can all be brought on by type gamma reactions. They are also known as “immunological imbalance syndromes and cytokine syndromes”.

Cross-reactivity reactions known as type delta reactions can happen when targeted antigens are expressed on different cells or structures. Adverse effects that are not immunologic are known as type epsilon responses. The classification system developed by [578] shows that even with the progress made from murine antibodies to human/humanized mAbs and the corresponding decrease in immunogenicity, adverse outcomes remain likely. Recombinant mAbs relate to certain dangers as biological products, although they do not carry the same infectious issues as products made from human donor blood. This class of drugs is also linked to a subset of other factors that need to be considered during prescription.

### Monoclonal antibodies-related limitations and adverse effects

Assuring the patient does not have latent tuberculosis (TB) is a necessary extra step while using TNF-a inhibitors like adalimumab. TNF-a signaling keeps the germs contained in the granulomas that form during tuberculosis infections. The bacteria's containment is disturbed, and latent infection is reactivated after TNF-a is inhibited (for example, by the delivery of mAbs). Similarly, biologics work by reducing the immune system of the host and consequently weakening the body's defenses, which may put patients at risk for sepsis, atypical infections (like fungi), and opportunistic infections. Prescribers must be aware of these consequences and ready to handle any adverse events that may arise from using these drugs. Reactions associated with infusions usually happen during or shortly after the delivery of mAb infusions, though they might happen afterward [579].

These are acute reactions associated with the host immunological response, immunogenicity, or intended pharmaceutical activity. Although the precise processes underlying these reactions are not well known, it is believed that complement cascade activation and the production of pro-inflammatory cytokines are involved. Reactions associated with infusions fall into type alpha reactions, which include localized injection site reactions, cytokine release syndromes (which include, in moderate cases, tachycardia, fever, dyspnea, and nausea), and, in severe situations, cytokine storm. The intensity of reactions will determine the best course of action, just like with other infusion-related events. The offending medication must be stopped in cases of severe responses including multiple organ failure, and supportive measures (such as mechanical breathing, IV fluids, vasopressors, HD, etc.) must be started [580].

In moderate situations, it may be possible to briefly stop the infusion and administer supportive drugs (paracetamol and diphenhydramine, for example) before gradually resuming the infusion. One type 1 acute hypersensitivity reaction that can also happen when using mAbs is anaphylaxis. Anaphylaxis is the result of IgE antibodies developing against mAbs, and it is usually not anticipated to occur on the recipient's first encounter with a particular mAb. This is because host IgE cross-reactivity has been reported, yet sensitization to the mAb cannot occur without prior exposure. According to the classification system put forth by [578], anaphylaxis is categorized as a kind of beta reaction [579]. Anaphylaxis brought on by mAbs can be treated in the same way as anaphylaxis brought on by other drugs. Similarly, with other drugs, each specific mAb may have unanticipated side effects that are directly connected to its intended mode of action (type A responses).

The use of abciximab, a mAb against glycoprotein (GP) IIb/IIIa, in patients having percutaneous coronary intervention (PCI) that results in significant bleeding events is one example of how mAbs are useful [581]. In patients with coronary artery disease, antiplatelet drugs are used to reduce the risk of stent thrombosis, periprocedural problems, and related ischemic events. By binding to the GP IIb/IIIa receptor and inhibiting it, abciximab stops platelet aggregation. By the same mechanism that makes it effective in stopping platelet aggregation, this monoclonal antibody may cause significant bleeding episodes in individuals who receive it [581].

### Bispecific monoclonal antibodies-related limitations and adverse effects

Regrettably, many of the disorders for which monoclonal antibodies are used have complicated pathophysiologies that are not caused by a single molecule or mechanism [582, 583]. The development of bsAbs presents a potential solution to this conundrum, as the idea of targeting several pathophysiological processes may increase therapeutic efficacy while also increasing the possibility of side effects [584]. The literature primarily describes four areas for the development of bsAbs: recruitment of Fc receptor-deficient T-cells (which would not normally be activated by antibody stimulation because of the absence of Fc receptors), inhibition of multiple cell surface receptors, multiple ligand blockade, and receptor cross-linkage. Though the assertions are essentially valid, designing and implementing bsAbs has proven to be difficult.

One such medication, catumaxomab, was first licensed in 2009 for the treatment of malignant ascites; however, despite its success, it was taken off the market in 2017 due to unfavorable events. It has been said that the mechanism of action is trifunctional. The molecule has an Fc region as well as two antigen-binding domains. Malignant cells have a trans-membrane glycoprotein called EpCAM, which is the target of the first antigen-binding area. The immune system's T-cells' CD3 antigens are bound by the second antigen-binding region. To combat cancerous cells, the Fc region serves to mobilize and attract immune cells such as natural killer cells and macrophages. All things considered, catumaxomab triggers several pathways for cytokine-mediated cytotoxicity and immune-mediated cell death (such as ADCC, ADCP, etc.) [585].

## Prospective insights on incorporating artificial intelligence (AI) to accelerate and redirect mAbs development and applications

### AI-based automation of the different steps of mAbs discovery

#### Epitope mapping

Epitope and paratope prediction, which entails predicting the regions of each protein involved in their interaction, the region on the antigen side is called an epitope, and the region on the antibody side is called a paratope, has been the first application of AI in the context of antibody discovery. The problem was initially tackled by predicting only linear epitopes, which account for only 10% of antibody epitopes [586, 587]. However, as more sophisticated algorithms were gradually introduced, including docking and machine-learning-trained scoring functions, useful accuracy levels were achieved [588–592]. Numerous examples [593–599] demonstrate the successful application of these strategies.

#### Screening clones

The first set of hits is primarily selected on the recombinant target using classical biology approaches based on hybridomas or display technologies, either in bacteriophages or yeasts [600], regardless of whether working from immune animals or already established antibody banks. The primary condition for success is high affinity. There are three main shortcomings to this approach: (1) The threshold of such procedures excludes many leads exhibiting sub-optimal affinity or less represented molecules, and (2) the epitope cannot be selected, indicating that selected hits attach to diverse locations on the target molecule. It is far from straightforward to experimentally identify the epitopes of these hits, or at least to know which ones compete (epitope binning); (3) the mechanism by which the animal immune repertoire is transferred to either bacteriophages or yeasts. The resultant antibodies are mostly non-natural because heavy and light chain pairing is not preserved.

The procedure of this first clone selection has been substantially enhanced more recently by single B cell technology [601]. Using single-cell technology, the animal's B-cells, which each produce a distinct antibody in their membrane, can be directly picked based on their affinity for the target, as opposed to creating a bank from the immune repertoire. Natural-paired sequences can then be produced by sequencing each of the antibodies that the B-cells were kept coded for separately. But this method also depends on high-affinity selection, which means that molecules with less representation or those with suboptimal affinity are again removed. Furthermore, experimental characterization still presents a challenge within a few thousand clones. As of right now, no documented in silico technique enables lead discovery against a chosen target while thoroughly examining the sequential space of a naturally occurring repertoire that is varied on both frameworks and CDRs.

Even with these advanced techniques, the search is still guided by a seed antibody. A future expansion of antibody repertoires is made possible by the impressive success deep-learning language models have had in identifying new and improved leads in enormous artificial libraries of CDR-degenerated parental antibodies [602–605].

#### Affinity evaluation and optimization

Since affinity evaluation is a relatively high throughput experimental technology compared to other in vitro experiments, it is frequently the first step in antibody characterization. While more accurate but low throughput evaluation is carried out in surface plasmon resonance (SPR) to provide the ground-truth dissociation constant (K_D_), rough but large-scale evaluation is frequently carried out in ELISA. However, the quantity of clones that may be assessed is limited by these technologies, as they necessitate the creation of both antigens and antibodies. Therefore, affinity prediction based on antigen and antibody sequences and structures would enable the assessment of considerably bigger ensembles. For this goal, numerous computational approaches and benchmarks have been put forth; yet the models' effectiveness is still restricted [606].

Furthermore, a lot of these techniques depend on the precise structural assembly of targets and antibodies, which is typically not known and especially not for large antibody collections. Immunization or screening of pre-existing antibody banks can provide antibodies with inadequate affinities. Extensive wet lab work is needed for experimental approaches to increase affinity, which rely on random mutagenesis and are often limited to the CDRs. Deep learning language models have demonstrated their ability to identify superior binders compared to parental antibodies. Language models begin by constructing a library of the parental antibody, in which CDR residues are degraded and substituted in all 20 or a subset of amino acids, using the same basic idea as the experimental method [605].

Nevertheless, the theoretical variety to investigate, even when solely considering the CDRs, remains much outside the scope of any wet-lab or computational approach, and maturation techniques are limited to considering a small number of altered sites. In terms of dimension, evaluating the entire theoretical mutational space of the CDRH3 only increases the library to 10^20^, given that it is 10 aa-long on average. By cloning degraded trastuzumab libraries in either bacteriophages or hybridomas and using their binding to a fluorescent HER2 (in FACS) to train models, Clark et al*.* 2023 were able to recover better binders than the parentals [605]. On positions 10 and 17, respectively, they included up to three mutations. Saka et al*.* 2020 [602] and Liu et al*.* 2021 [604] constructed degenerated libraries of anti-VEGF-A and anti-kynurenine, respectively, and utilized the enriched sequences to train a directed evolution-based model during panning cycles.

Aside from the limited number of mutations, the main drawback of these models is that they are trained on a specific antibody-antigen pair, meaning the training set that is produced is not target-agnostic. The following target is not covered by the entire process. The success rate of affinity maturation has grown dramatically with the logical design of mutants due to advancements in structural identification technologies [607]. While rational design demands precise structural data, which is a challenging undertaking in and of itself, it also results in testing a significantly smaller number of mutants than random mutagenesis. Numerous computational techniques that enable the prediction of mutant affinity have recently emerged [607] to address this issue, with varying degrees of effectiveness [608].

#### Off-target prediction

Off-target binding is one aspect that is frequently disregarded when searching for antibodies. Binding to unrelated proteins is typically ignored or only considered relatively late in the discovery phase if selectivity for the target is confirmed by assessing the lack of binding to close homologs. However, a wealth of evidence now suggests that this phenomenon, known as cross-reactivity, is far from anecdotal, as it is likely to be the cause of some clinical trial failures and a contributing factor to auto-immune diseases [609–612]. Cross-reactivity, however, might also work to your benefit, as rituximab demonstrates. There are certain expensive and time-consuming experimental techniques for assessing cross-reactivity, such as protein arrays and tissue cross-reactivity. Recently, a computational technique has been developed that enables us to accurately forecast off-target binding [613].

This technique encodes the CDRs of the antibodies using both the sequence and the expected 2D structure of the antibodies. Based on the similarity of item sets, these encodings can then be compared using a particular score [614, 615]. When this technique is applied to conjugation with databases of antibodies that have identified targets, it can detect off-targets as soon as the sequences are known.

#### Developability prediction and optimization

The assessment of developability is the final phase in the antibody discovery process. Developability refers to a variety of factors: (1) Immunogenicity: when injected into humans, would this antibody cause an immunological response? (2) Producibility: in the bioproduction process, would this antibody have high yields of production? (3) Aggregation: Can a high-concentration solution be created, or will the antibody clump together? A thorough evaluation of the databases and techniques created thus far is provided [616]. To put it briefly, humanness scores have a major role in immunogenicity prediction [617]. These scores relate to the amount of anti-drug antibodies (ADA) seen in clinical trials and assess how closely the antibody of interest resembles known human sequences. Humanization, or altering patterns to return to the closest human germline, is the first step in optimizing an antibody's immunogenicity. The CDR similarity metric makes humanization possible.

It is possible to argue that the frameworks of the retrieved human antibodies provide the best scaffolding for supporting the CDRs since they can be used to locate the human antibody with the most similar CDRs. A fully human candidate can subsequently be achieved by grafting the animal antibody's CDRs into human frameworks. The therapeutic antibody profiler (TAP) tool provides a broader assessment of developability [618]. This technique makes it possible to predict how an antibody will express itself or aggregate depending on properties like the length of the CDRH3 and the hydrophobicity of the CDRs or canonical forms. Interpretable neural networks are used by [619] to accurately predict melting temperature and aggregation. A software, which is based on a random-forest method and employs both sequence and structure, was created by [620].

Predicting producibility appears to be an even more challenging task. Several studies [621, 622] demonstrate a relationship between the production titer and the stability of the antibody, particularly the melting temperature and solubility. Convolutional neural networks and pre-trained language models are used by [623] to forecast melting temperature. While avoiding antibodies with low melting temperatures or poor solubility is preferable, it does not ensure high-quality manufacturing titers.

### Honing and chaining all steps through AI-based de novo mAbs discovery

By starting with the target's name alone, de novo antibody design aims to produce an extremely affine, soluble, non-immunogenic, and epitope-directed antibody. It requires at the very least mastery of structural characterization, developability evaluation, and affinity prediction. As previously noted, solutions designed to address each potential hazard are devised, but they are nonetheless applied one at a time along the funnel-shaped process that is governed by the traditional biological pipeline. Creating a moral circle by connecting them all is undoubtedly one of the keys to success. The first step in the entire design process needs to be the creation of candidates, either arbitrarily, which would indicate a sensible selection procedure later, or logically, by "walking" on the intended structure.

The first method was anticipated to be fulfilled at high throughput by language-based approaches; however, as previously said, they are still severely constrained in the diversity of antibodies that can be inserted into the computations [604, 624–626]. Aguilar Rangel et al*.* method [627] computes CDR and epitope peptide complementarity using a structural approach. The technique, according to the authors, can create de novo CDR peptides that, albeit with restricted affinities, can be grafted onto nanobodies that bind to three distinct targets: trypsin, SARS-CoV-2 spike protein, and human serum albumin. Although it hasn't been used for antibodies yet, Anishchenko et al*.* technique [628], which computes a structural evaluation of randomly produced and modeled peptides, has proven accurate for protein design.

## Conclusion

This review comprehensively presented an overall view of the background, fundamentals, and future of mAbs development platforms, techniques, and technologies with a deep and wide glimpse of the mAbs therapeutic applications either in both infectious and noninfectious diseases. In this context, mAbs can potentially provide more effective and safer treatment options than traditional therapies. In a world of antibiotic-resistant superbugs and an aging population grappling with autoimmune disorders and cancer, mAbs offer the potential for new, targeted treatments and drugs that can provide personalized care, and a window into the complex, overlapping conditions that underlie human disease.

Despite the tremendous progress in developing new mAbs with a wide range of developmental technologies and the significant advantages that have been achieved using previously developed mAbs in the field of medicine that have revolutionized the treatment of various diseases, there are still many obstacles to overcome in every step of the developing process, as well as clinical and market challenges that make them commercially less attractive which includes the high cost of production, and administration challenges may hinder their widespread accessibility, immunogenicity, and limited target range are additional considerations that need to be addressed to optimize their effectiveness. Many efforts are required to overcome these challenges, including advancing more potent mAbs, development of new formulation and delivery methods, efficient clinical trials that include mAb combinations, and engagement with organizations operating in low- and middle-income countries to favor technology transfer and access to these new bioproducts.

The ability to engineer these molecules to improve their properties and target intracellular compartments, bind two different antigens simultaneously, deliver drug conjugates, and generate Fc fusions revolutionized the treatment of diseases. Out of more than 100 currently approved mAbs, 6 were approved in the 1990s, 16 from 2000 to 2010, 70 from 2011 to 2020, and 32 in the past three years (2021–2023), thus showing an upward growth in the production and marketing authorization of therapeutic mAbs. Also, AI has the potential to accelerate mAb development, enhance production processes, improve therapeutic efficacy, and enable personalized medicine approaches. However, it's important to note that while AI can provide valuable insights and predictions, experimental validation and human expertise remain crucial for successful mAb synthesis and application. Finally, mAbs are providing a promising alternative and strategic therapeutic panel that grows day after day.

## Supplementary information

Supplementary Material 1. An illustrative structural diagram facilitates an understanding of the hierarchical structure of this review article.

## Data Availability

All data generated or analyzed exist in the submitted manuscript.
