# Supplementary material for: Monoclonal antibodies: From magic bullet to precision weapon
Source: Mol Biomed. 2024 Oct 11;5:47. doi: 10.1186/s43556-024-00210-1 (PMC11467159; doi:10.1186/s43556-024-00210-1)
Supplement: Supplementary file 1 — Supplementary Material 1. An illustrative structural diagram facilitates an understanding of the hierarchical structure of this review article. [file 43556_2024_210_MOESM1_ESM.pdf]

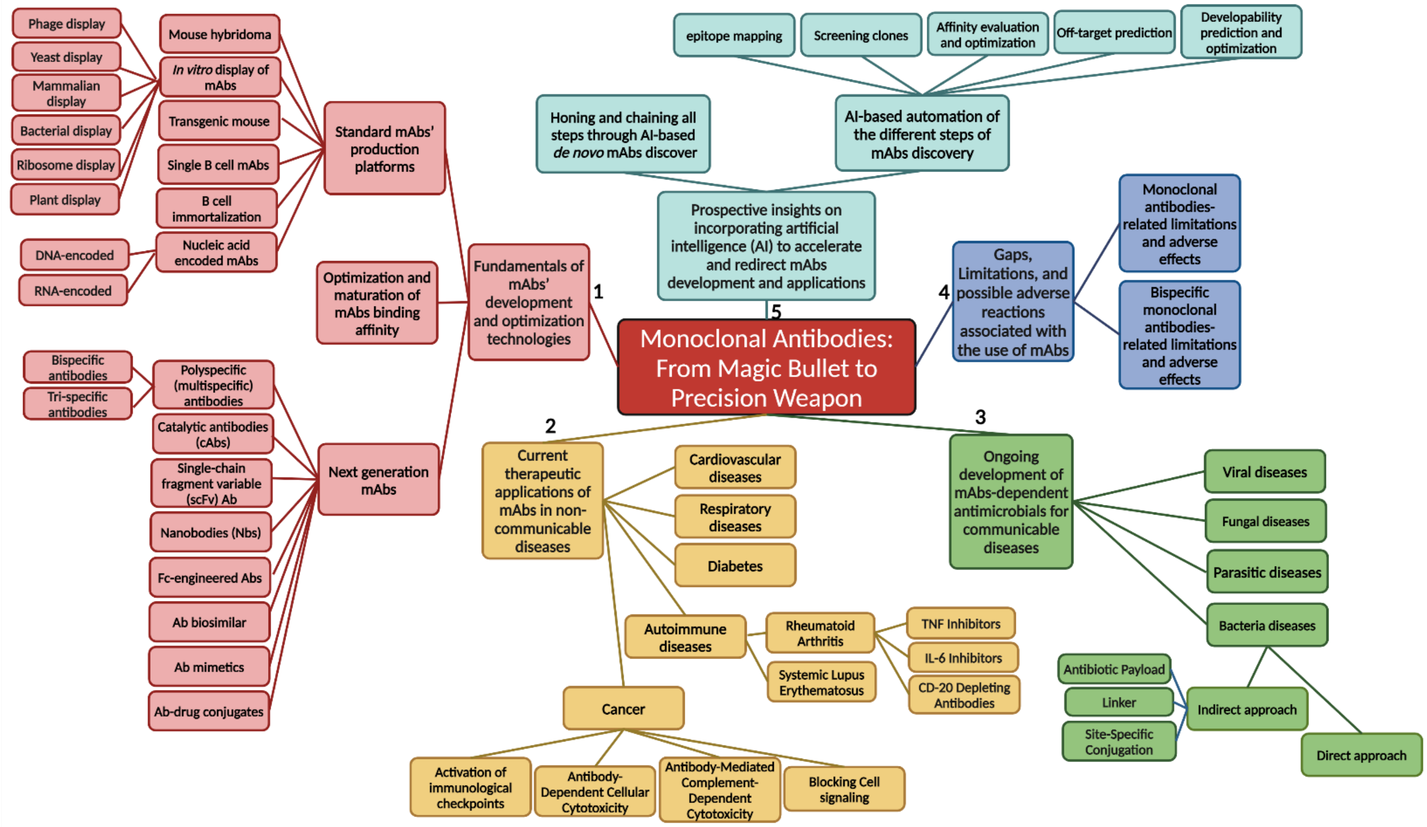

Supplementary Fig. 1: An illustrative structural diagram facilitates an understanding of the hierarchical structure of this review article.
